# Aberrant Herpesvirus-Induced Polyadenylation Correlates With Cellular Messenger RNA Destruction

**DOI:** 10.1371/journal.pbio.1000107

**Published:** 2009-05-26

**Authors:** Yeon J. Lee, Britt A. Glaunsinger

**Affiliations:** Department of Plant and Microbial Biology, University of California Berkeley, Berkeley, California, United States of America; University of Wisconsin-Madison, United States of America

## Abstract

Regulation of messenger RNA (mRNA) stability plays critical roles in controlling gene expression, ensuring transcript fidelity, and allowing cells to respond to environmental cues. Unregulated enhancement of mRNA turnover could therefore dampen cellular responses to such signals. Indeed, several herpesviruses instigate widespread destruction of cellular mRNAs to block host gene expression and evade immune detection. Kaposi's sarcoma-associated herpesvirus (KSHV) promotes this phenotype via the activity of its viral SOX protein, although the mechanism of SOX-induced mRNA turnover has remained unknown, given its apparent lack of intrinsic ribonuclease activity. Here, we report that KSHV SOX stimulates cellular transcriptome turnover via a unique mechanism involving aberrant polyadenylation. Transcripts in SOX-expressing cells exhibit extended poly(A) polymerase II-generated poly(A) tails and polyadenylation-linked mRNA turnover. SOX-induced polyadenylation changes correlate with its RNA turnover function, and inhibition of poly(A) tail formation blocks SOX activity. Both nuclear and cytoplasmic poly(A) binding proteins are critical cellular cofactors for SOX function, the latter of which undergoes striking nuclear relocalization by SOX. SOX-induced mRNA turnover therefore represents both a novel mechanism of host shutoff as well as a new model system to probe the regulation of poly(A) tail-stimulated mRNA turnover in mammalian cells.

## Abstract 2

Inhibition of host cell gene expression by the human herpesvirus KSHV occurs via a novel mechanism involving polyadenylation-linked RNA turnover.

## Author Summary

During viral infection, many essential cellular functions are targets for viral manipulation, yet aside from RNA interference, surprisingly few examples of viruses disrupting RNA turnover have been documented. Kaposi's sarcoma-associated herpesvirus (KSHV) is an oncogenic virus that induces widespread cellular messenger RNA destabilization during lytic infection. The viral protein SOX is a critical effector of this phenotype, yet it lacks ribonuclease activity, so presumably it targets cellular factors governing RNA stability. Here, we show that SOX stimulates host mRNA destruction via a unique mechanism involving polyadenylation. During SOX expression, newly formed messages have longer than normal poly(A) tails, leading to their retention in the nucleus. Coincident with this hyperadenylation, poly(A) binding protein (PABPC) is relocalized from the cytoplasm to the nucleus. PABPC has prominent roles in translation, messenger RNA stabilization, and quality control in the cytoplasm; we find its nuclear relocalization by SOX correlates with enhanced mRNA turnover in the cytoplasm. Thus, KSHV appears to have evolved distinct polyadenylation-linked mechanisms to target both new messages in the nucleus and preexisting cytoplasmic messages for destruction, thereby effectively inhibiting cellular gene expression.

## Introduction

Kaposi's sarcoma-associated herpesvirus (KSHV) is the most recently discovered human herpesvirus and the etiologic agent of several neoplasms, the most prominent of which is Kaposi's sarcoma (KS) [1]. Originally described as a rare tumor found predominantly in elderly Mediterranean or African men, with the onset of the AIDS pandemic, KS became the most common neoplasm associated with untreated human immunodeficiency virus (HIV) infection. KSHV is a large double-stranded DNA virus that undergoes both latency and lytic replication. Although the majority of infected cells in vitro and in vivo harbor the virus in a latent state, the lytic cycle is required both for viral replication and KS development [2],[3]. One striking feature of lytic KSHV infection is that it destroys the host transcriptome by promoting global messenger RNA (mRNA) degradation via unknown mechanisms [4],[5]. The magnitude of cellular transcript loss is significant; nearly 75% of all messages are massively down-regulated, with another 20% undergoing a more modest decrease [6],[7]. This phenotype, termed host shutoff, is mediated by the viral factor SOX (shutoff and exonuclease) which has homologs across the entire herpesvirus family [5]. In other herpesviruses, this protein has DNA exonuclease (DNase) and recombinase activities that contribute to processing and packaging the newly replicated viral genomes in the nucleus, but has no role in mRNA turnover [8]–[10]. By contrast, in KSHV and its closest viral relatives within the lymphotrophic γ-herpesviral subfamily—including the human cancer-associated Epstein-Barr virus—SOX retains these conserved functions but has evolutionarily acquired a novel and distinct role in global mRNA decay [11],[12]. The host shutoff and DNA processing functions of SOX are genetically separable, as single-function point mutants can dissociate the two activities [4]. Despite its ability to induce widespread mRNA destruction, KSHV SOX has neither homology to known ribonucleases nor predicted RNA recognition motifs, and thus far no intrinsic ribonuclease (RNase) activity has been detected with the purified protein. SOX is therefore presumed to function by modulating one or more cellular RNA turnover pathways.

Control of message stability obviously represents a powerful means of regulating gene expression both on an individual and a global scale. Nearly all eukaryotic mRNAs are protected from exonucleolytic attack by a 5′ cap structure and a 3′ poly(A) tail. Cleavage and polyadenylation are cotranscriptional events, and their successful completion is required to signal formation of an export competent message. Poly(A) site recognition is mediated by specific sequence elements bound by the cleavage factors CPSF, CtsF, and CFI_m_ [13]–[15]. Poly(A) polymerase (PAP) is recruited to the complex during the cleavage reaction and initiates polymerization of the adenosine tract in a biphasic manner; initial slow distributive adenosine addition proceeds until a sufficiently long tail has been formed to allow binding of nuclear poly(A) binding protein (PABPN), then rapid polymerization of the remaining 200–250 nucleotides (nt) ensues, whereupon PAP reverts to a distributive mode and dissociates from the transcript [16]. Upon nuclear export, PABPN is replaced by the cytoplasmic poly(A) binding protein (PABPC), which enhances mRNA stability and translation efficiency, in part through its interactions with the eIF4G translation initiation factor [17],[18].

In eukaryotes, polyadenylation generally serves to stabilize mRNAs, whereas in bacteria and some organelles, it facilitates RNA turnover [19]. However, it is now becoming clear that polyadenylation can be a facilitator of eukaryotic mRNA degradation as well. In particular, yeast possess a nuclear polyadenylation complex (TRAMP) that marks aberrantly processed RNAs for quality control-mediated turnover via the addition of short poly(A) tails [20]–[22]. Additionally, many yeast mutants defective in RNA processing or export accumulate hyperadenylated transcripts, suggesting a link between the polyadenylation process and RNA surveillance and turnover [23]–[25]. Analogous pathways may function in higher eukaryotes, as polyadenylated precursors to RNA turnover have also been detected in mammalian cells [26]–[28].

Here, we reveal that KSHV SOX-induced host shutoff is intimately linked to mRNA polyadenylation. SOX promotes aberrant polyadenylation of cellular transcripts in a manner dependent on its RNA turnover activity. Transcript degradation requires both nuclear and cytoplasmic poly(A) binding proteins, the latter of which undergoes striking nuclear relocalization in a host shutoff-dependent manner. In the absence of cellular 3′ end processing and polyadenylation, SOX can no longer target mRNAs for destruction, although addition of a templated poly(A) tail reinstates SOX-induced turnover. These findings suggest SOX is directing a novel polyadenylation-dependent mechanism of host shutoff, and demonstrate a link between polyadenylation and mRNA destruction in higher eukaryotes.

## Results

### KSHV SOX Promotes Hyperadenylation of Cellular mRNAs

**Figure 1:**
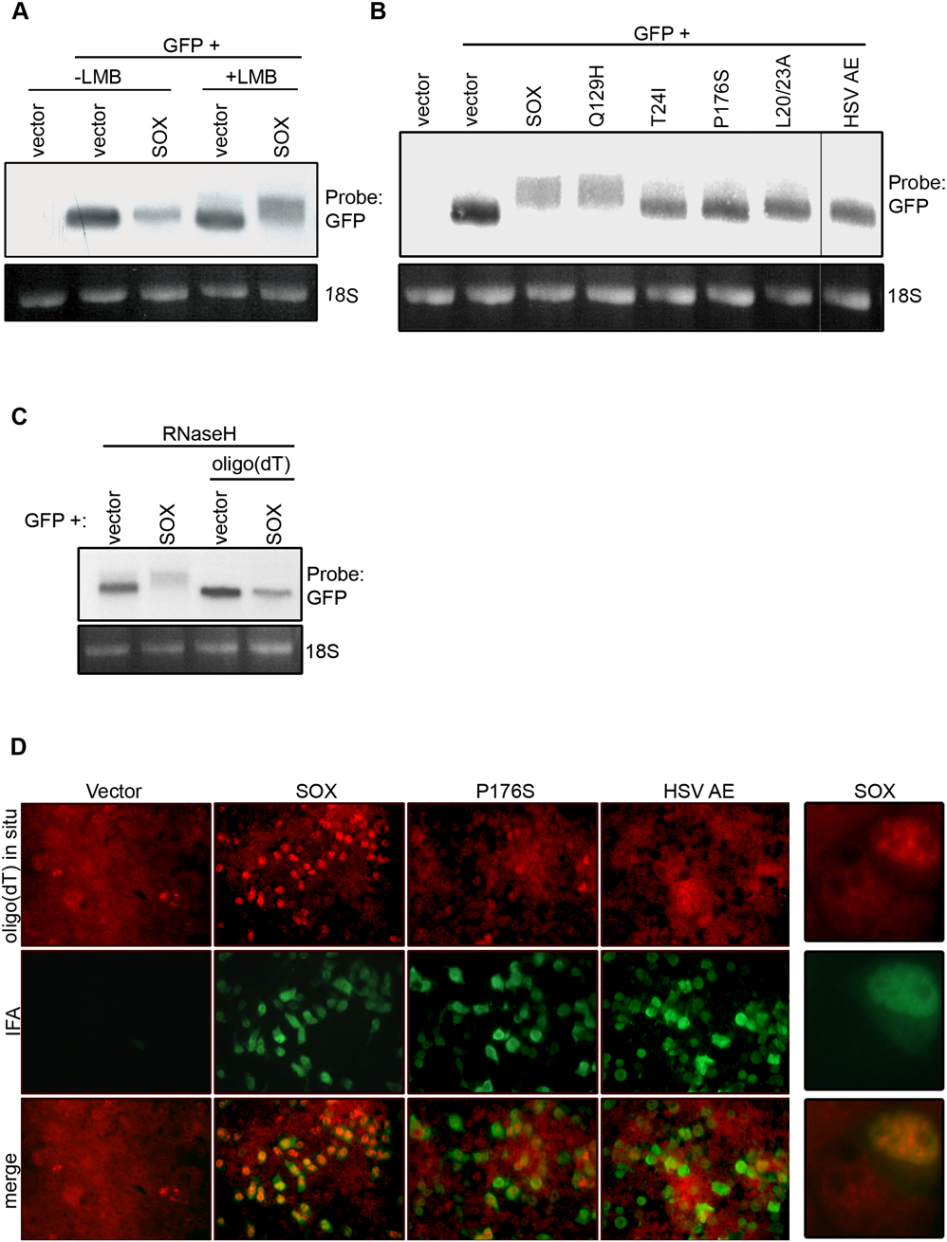
SOX induces mRNA hyperadenylation in a manner dependent on its RNA turnover activity. (A and B) HEK 293T cells were transfected with a plasmid expressing GFP alone or together with a plasmid expressing SOX and either left untreated (A) or treated with 5 ng/ml LMB for 6 h (A and B). Twenty-four hours post-transfection, total RNA was harvested, resolved on an agarose-formaldehyde gel, and northern blotted with a ^32^P-labeled GFP probe. The line through the gel indicates where an intervening lane was cropped out of the image (B). (C) HEK 293T cells were transfected with the indicated plasmids and treated with LMB as described above. Total RNA was prepared from the cells and digested with RNaseH in the presence or absence of oligo(dT), then resolved by agarose-formaldehyde electrophoresis, and then northern blotted with a ^32^P-labeled GFP probe. In (A–C) 18S rRNA serves as a loading control. (D) HEK 293T cells were transfected with the indicated plasmid and, 24 h later, subjected to oligo(dT) in situ hybridization (upper panels) followed by staining with SOX antibodies (for vector, SOX, P176S samples) or HA antibodies (for HA-HSV AE sample) (center). The lower panels show overlap of the oligo(dT) and antibody staining. The right column shows a magnified version of two cells (one expressing SOX and one lacking SOX) from an inset derived from the SOX-transfected sample.

The KSHV SOX protein is found both in the nucleus and in the cytoplasm of cells, whereas its herpesviral homologs lacking mRNA turnover activity are restricted to the nucleus [4]. We considered that this distinct localization pattern could play a role in its mRNA degradation function, and therefore evaluated whether blocking CRM1-dependent nuclear protein export using the drug leptomycin B (LMB) could restrict SOX to the nucleus and alter its function. Although LMB treatment significantly increased the population of SOX in the nucleus (Figure S1), it did not completely eliminate the cytoplasmic fraction and did not abrogate the mRNA turnover activity of SOX (Figure 1A–1C). However, upon LMB treatment, a slower-migrating population of the reporter green fluorescent protein (GFP) mRNA appeared specifically in the presence of SOX, indicative of some form of SOX-induced RNA modification (Figure 1A). To gain insight into the specificity of this SOX activity, we tested a panel of SOX mutants lacking only the conserved DNase activity associated with viral genome processing or lacking only the mRNA turnover activity responsible for host shutoff [4],[5]. The production of these slower-migrating species correlated very strongly with the host shutoff function of SOX; they were not observed in cells expressing SOX mutants defective for mRNA degradation (T24I, P176S, L20/23A), but they were produced in cells expressing a SOX mutant (Q129H) lacking only the conserved DNase activity (Figure 1B). Furthermore, expression of the SOX homolog from herpes simplex virus (HSV AE) that exhibits DNase activity [8],[29], but has no role in host shutoff [5], also has no effect on the reporter mRNA mobility (Figure 1B). Thus, we conclude that this RNA modification correlates with the RNA turnover activity of SOX responsible for host shutoff.

One obvious mRNA modification that could significantly alter message size is polyadenylation. To determine whether the altered mRNA mobility was due to extended poly(A) tails (hyperadenylation), we investigated whether deadenylation of the messages by oligo(dT) hybridization followed by RNaseH digestion would eliminate their size differences. Indeed, northern blotting revealed that poly(A) tail removal caused the high MW mRNA from SOX-expressing cells to shift down in size, such that it precisely co-migrated with the ‘normal’ mRNA (Figure 1C).

Although LMB treatment may somehow stabilize the hyperadenylated mRNA species thereby facilitating their detection by northern blotting, it was important to confirm both that this modification also occurs in untreated cells and on endogenous cellular transcripts. To this end, total endogenous poly(A) RNA accumulation was measured by in situ hybridization of HEK 293T cells with a fluorescently labeled oligo(dT) probe (Figure 1D). Significantly, all wild-type (WT) SOX-expressing cells contained elevated levels of endogenous nuclear poly(A) RNA, as visualized by enhanced oligo(dT) staining. Accumulation of the poly(A) RNA specifically in the nucleus can be seen most clearly in the higher magnification images (Figure 1D, far right). Biochemical fractionation studies also show that the hyperadenylated mRNA is absent from the cytoplasmic fraction of cells (Figure S3). SOX single-function mutants lacking host shutoff activity, such as P176S and the HSV SOX homolog (AE) that possesses only DNase activity, fail to increase cellular poly(A) RNA levels (Figure 1D and unpublished data). These observations indicate that hyperadenylation is widespread on endogenous messages in SOX-expressing cells. Thus, although polyadenylation has traditionally been viewed as a stabilizer of eukaryotic transcripts, our data indicate that it is associated with mRNA destruction in the presence of SOX.

### Poly(A) Polymerase II Mediates Hyperadenylation in SOX-Expressing Cells

**Figure 2:**
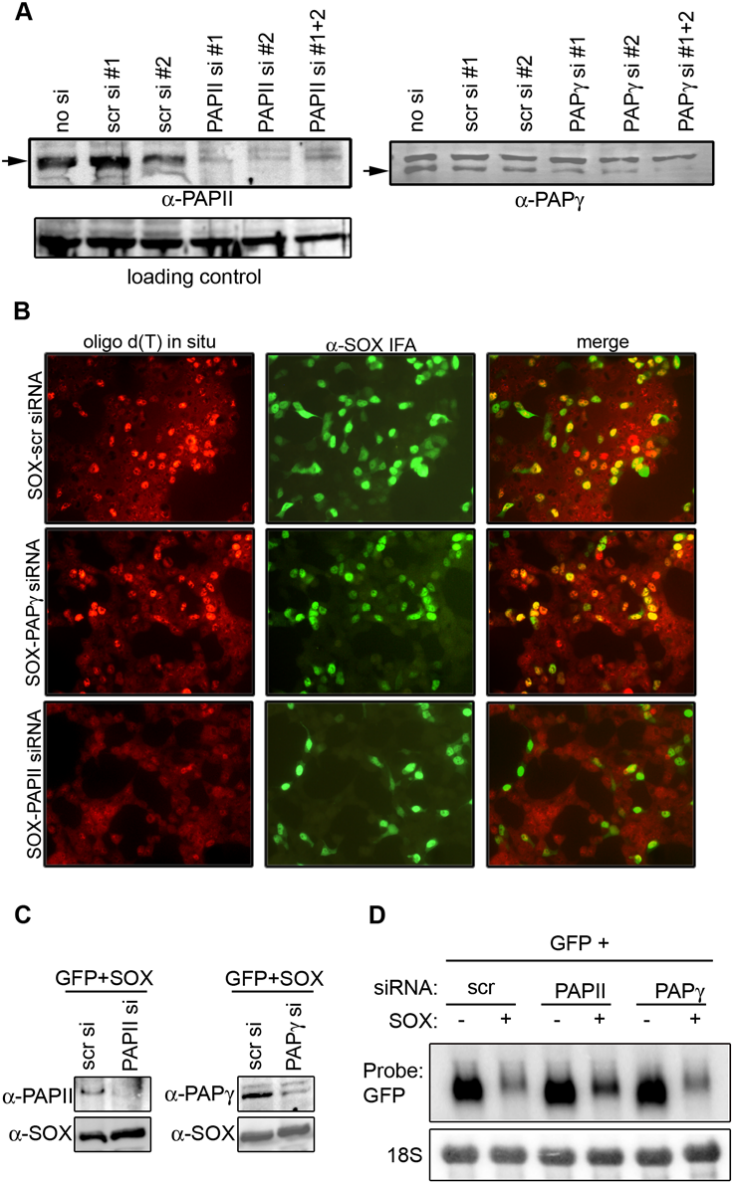
SOX-induced cellular poly(A) RNA accumulation occurs via PAPII. (A and B) HEK 293T cells were either mock transfected or transfected twice with PAPII or PAPγ duplex siRNA oligos (si #1, si #2, or a mixture of #1+#2) or one of two nonspecific control siRNA oligos (scr si #1 or scr si #2). Twenty-four hours after the final siRNA transfection, the cells were transfected with a DNA plasmid expressing SOX; each sample was split in half and, 24 h later, either harvested for protein and immunoblotted with PAPII and PAPγ antibodies to gauge the efficiency of siRNA-mediated knockdown (A), or processed for oligo(dT) in situ hybridization and α-SOX immunofluorescence to monitor the efficiency of SOX-induced poly(A) RNA accumulation (B). Arrows denote the location of PAPII and PAPγ protein on the western blots in (A). Nonspecific cross-reactive bands serve as loading controls. (C and D) HEK 293T cells were transfected with the indicated siRNA as described above, followed by subsequent transfection with either a plasmid expressing GFP alone or together with a SOX expression plasmid. Cells were treated with 5 ng/ml LMB for 6 h prior to harvesting either protein for western blotting with PAPII, PAPγ, and SOX antibodies (C), or RNA for northern blotting with GFP and 18S probes (D).

Three poly(A) polymerase proteins with molecular masses of 90, 100, and 106 kDa have been identified in HeLa cell nuclear extracts [30]. The 106-kDa isoform is likely a phosphorylated version of the 100-kDa isoform, and collectively, they are referred to as PAPII, whereas the 90-kDa protein, termed PAP-γ, is the product of a distinct locus [31]. A testes-specific PAP has also been identified [32],[33], but was not examined here due to its tissue-restricted expression. The contribution of PAPII and PAPγ towards SOX-induced hyperadenylation was assessed using small interfering RNA (siRNA)-based knockdown of each protein and measuring the resulting effects on endogenous poly(A) RNA accumulation by oligo(dT) in situ hybridization (Figure 2). Western blotting confirmed efficient knockdown of PAPII and PAPγ upon transfection of two independent siRNA oligo pairs (Figure 2A). We consistently observed that SOX-induced hyperadenylation was diminished in the absence of PAPII, whereas no decrease in the oligo(dT) signal in SOX-expressing cells was detected upon PAPγ knockdown (Figure 2B). Thus, hyperadenylation in SOX-expressing cells is mediated by the canonical PAP responsible for the majority of mRNA poly(A) tail synthesis. If hyperadenylation by PAPII participates in mRNA turnover by SOX, we would predict that inhibition of PAPII might stabilize mRNAs in SOX-expressing cells. We therefore monitored GFP mRNA turnover by SOX upon siRNA-mediated knockdown of PAPII or PAPγ (Figure 2C). Indeed, PAPII knockdown increased GFP mRNA levels in the presence of SOX, as well as increased the mobility of the GFP mRNA in the presence of SOX (Figure 2D, compare lane 4 to lanes 2 and 6; quantification shown in Figure S2). By contrast, GFP mRNA was still efficiently degraded and hyperadenylated upon knockdown of PAPγ. These data therefore suggest that PAPII-induced hyperadenylation plays an important role in SOX-induced host shutoff.

### SOX Promotes Nuclear Relocalization of Cytoplasmic Poly(A) Binding Protein

While PAPII mediates poly(A) tail formation, its activity is greatly stimulated by the nuclear poly(A) binding protein PABPN, which has also been proposed to help mediate poly(A) tail-length control [16]. Once synthesized, poly(A) tails are immediately coated with PABPN, which remains bound to the mRNAs until their transport into the cytoplasm, whereupon PABPN is replaced by cytoplasmic PABP (PABPC). PABPC effectively circularizes mRNAs by virtue of its interaction with eIF4G, an event that both protects the mRNA ends from exonucleolytic attack and enhances message translation via the closed loop model [17],[18]. Although both bind poly(A) tails, PABPN and PABPC have distinct functions and subcellular localizations and in fact do not share significant sequence homology.

**Figure 3:**
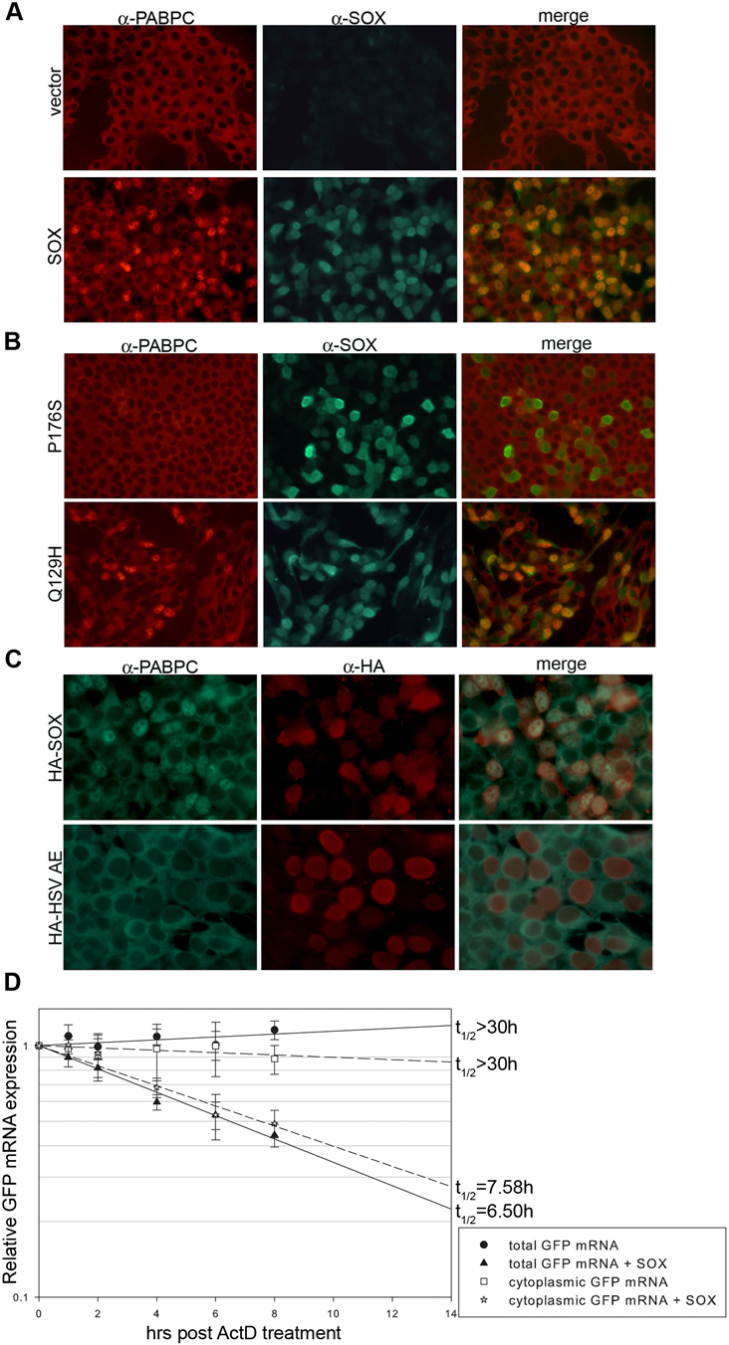
The host shutoff activity of SOX induces nuclear accumulation of PABPC and turnover of cytoplasmic mRNAs. (A) HEK 293T cells were transfected with empty vector or with a plasmid expressing SOX and, 24 h later, subjected to double-label immunofluorescence analysis with monoclonal 10E10 PABPC antibodies (left) and SOX polyclonal antisera (center). The overlap of PABPC and SOX staining can be viewed in the right column. (B) HEK 293T cells were transfected with the indicated single-function SOX mutant and subjected to immunofluorescence as described above using 10E10 PABPC antibodies and SOX antisera. (C) HEK 293T cells were transfected with HA-tagged WT SOX or the HSV AE SOX homolog and subjected to immunofluorescence as described above using polyclonal PABPC antisera and monoclonal HA antibodies. (D) HEK 293T cells were transfected with a plasmid expressing GFP alone or together with a SOX expression plasmid and, 24 h later, treated with 5 ng/ml LMB for 12 h. The cells were then incubated in media lacking LMB but containing 1 µg/ml actinomycin D to block transcription, and the cytoplasmic fraction was isolated at the indicated times. RNA was then northern blotted with GFP and 18S probes, and the half-life (t_1/2_) of the cytoplasmic GFP mRNA with and without SOX was calculated. Error bars show the standard error between samples. The graph represents a compilation of three independent experiments.

Given the strong associations between the PABPs and poly(A) tail formation, length control, and mRNA stability, we hypothesized that one or both of these proteins could be involved in the poly(A)-dependent mRNA turnover by SOX. We began by monitoring the localization of these proteins in cells with or without SOX. Remarkably, immunofluorescence experiments revealed that whereas PABPN localization was unchanged by SOX (unpublished data), there was a striking redistribution of endogenous PABPC from the cytoplasm to the nucleus (Figure 3A). This phenotype was confirmed using two independent PABPC antibodies (Figure 3A and 3C). Interestingly, we have not observed an interaction between SOX and PABPC in co-immunoprecipitation experiments from cells transfected with SOX or lytically infected with KSHV (unpublished data), suggesting that PABPC relocalization is not due to direct binding and recruitment by SOX.

Although PABPC is a nuclear–cytoplasmic shuttling protein [34] its steady-state localization is almost exclusively cytoplasmic and possible roles for PABPC in nuclear events such as mRNA 3′ end formation and quality control have not been elucidated. To link PABPC nuclear import mechanistically to SOX-induced mRNA turnover, we examined a panel of SOX mutants for their ability to redistribute PABPC. Single-function SOX mutants such as P176S and the HSV SOX homolog AE lacking the mRNA turnover and hyperadenylation functions failed to alter PABPC localization (Figure 3B and 3C). However, the Q129H SOX mutant that lacks the conserved DNase activity but retains the ability to promote host shutoff and hyperadenylation induced PABPC nuclear recruitment to the same extent as WT SOX (Figure 3B). Thus, nuclear accumulation of PABPC requires the host shutoff activity of SOX.

Removal of PABPC from the cytoplasm would be predicted to destabilize mRNAs in that locale. To test this hypothesis, we performed fractionation experiments to monitor the half-life of GFP mRNA specifically in the cytoplasm of cells with and without SOX. Indeed, we observed that cytoplasmic mRNAs were more rapidly turned over in SOX-expressing cells compared with cells lacking SOX (7.5 h versus >30 h) (Figure 3D; gels shown in Figure S3). The shortened half-life of cytoplasmic GFP mRNA was comparable to that of total GFP mRNA extracted from unfractionated cells expressing SOX (6.5 h) (Figure 3D).

**Figure 4:**
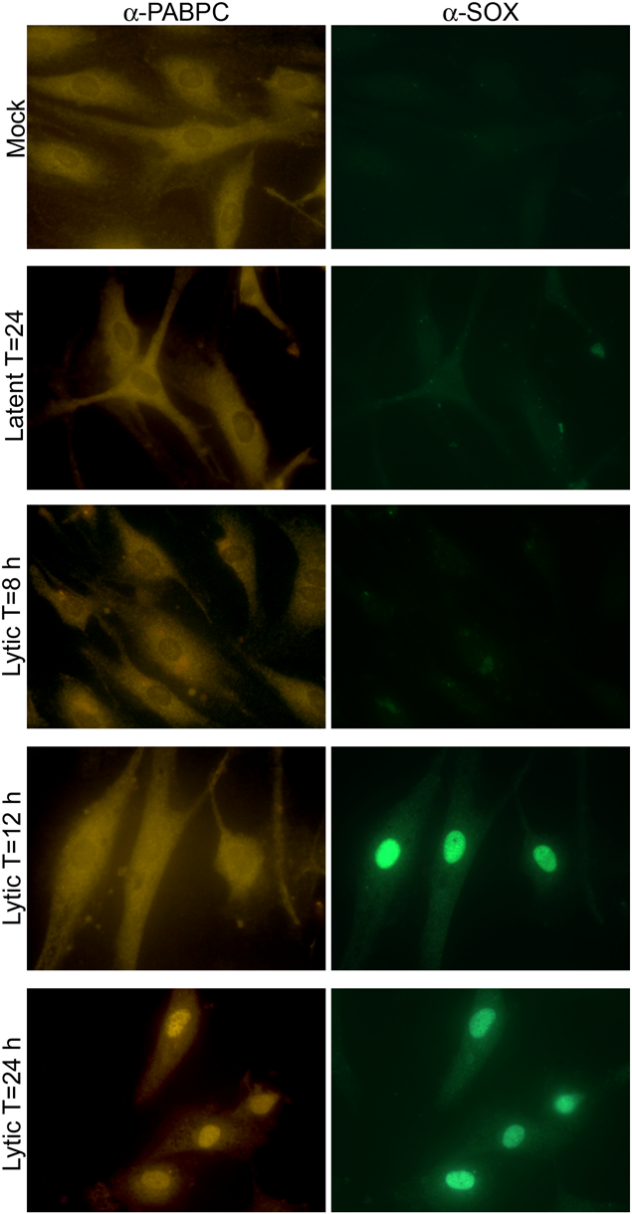
PABPC accumulates in the nucleus during lytic KSHV infection in a manner temporally coincident with host shutoff. TIME cells were either mock infected, latently infected with KSHV, or infected with KSHV and lytically reactivated using an adenoviral expression vector containing the viral lytic transactivator RTA (Ad-RTA) for 8, 12, or 24 h. Mock-infected cells were similarly treated with Ad-RTA. PABPC and SOX proteins were detected by immunofluorescence analysis with polyclonal PABPC antibodies and affinity purified SOX polyclonal antibodies.

SOX expression initiates 12 h into the KSHV lytic cycle, but cellular mRNA destruction becomes most prominent at 18–24 h and is maintained throughout the lytic cycle [5]. To monitor PABPC localization during infection, telomerase-immortalized microvascular endothelial (TIME) cells were either mock infected, latently infected with KSHV, or lytically infected with KSHV for a time course of 8–24 h. PABPC staining was predominantly cytoplasmic in mock-infected cells, as well as during latent infection and at 8 h post lytic infection when SOX is not expressed and host shutoff does not occur (Figure 4). However, beginning at the onset of host shutoff at 12 h post lytic infection, PABPC concentration in the nucleus began to increase, and by 24 h, the majority of infected cells showed prominent nuclear PABPC staining. These results confirm that PABPC relocalization into the nucleus is similarly induced during KSHV infection and is temporally coincident with SOX-induced host shutoff.

### Roles for Cytoplasmic and Nuclear Poly(A) Binding Proteins in SOX Function

**Figure 5:**
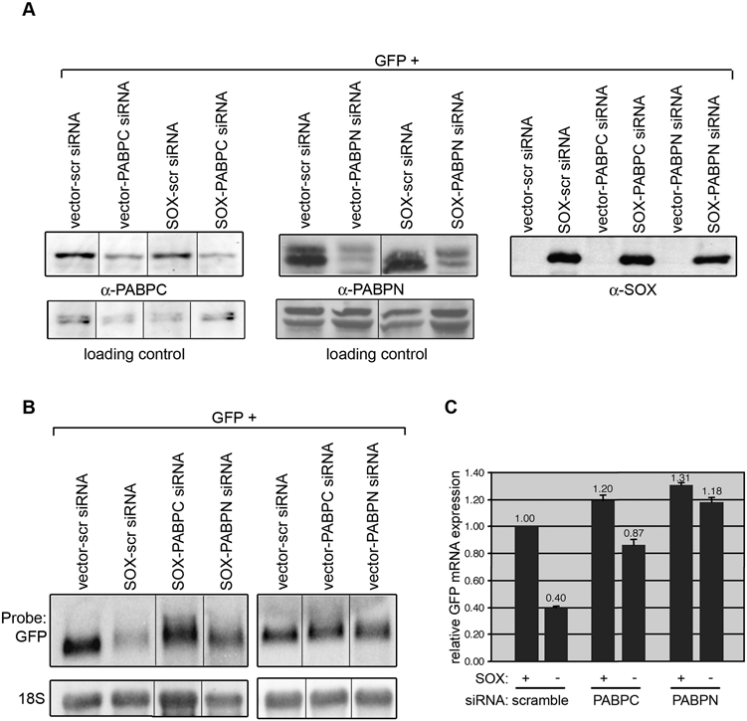
PABPC and PABPN are essential cellular cofactors for SOX-induced mRNA turnover. HEK 293T cells were transfected twice with PABPC or PABPN duplex siRNA oligos or a nonspecific control siRNA oligo (scr si). Twenty-four hours after the final siRNA transfection, the cells were transfected with a DNA plasmid expressing the GFP reporter alone or together with SOX; each sample was split in half and, 24 h later, either harvested for protein and immunoblotted with PABPC, PABPN, and SOX antibodies to gauge the efficiency and specificity of siRNA-mediated knockdown (A), or harvested for RNA and either northern blotted with GFP and 18S probes (B) or subjected to qPCR analysis (C) to monitor SOX activity. Each qPCR reaction was run in triplicate, and GFP mRNA levels were normalized to 18S RNA, because cellular housekeeping genes are subject to host shutoff by SOX. Error bars show standard error between sample replicates. Nonspecific cross-reactive bands serve as loading controls for the western blots in (A). Lines through gels indicate where intervening lanes were cropped out of the image.

The host shutoff-dependent nuclear redistribution of PABPC suggested that this factor could play a prominent role in mRNA turnover in SOX-expressing cells. Additionally, the fact that PABPN is functionally linked to poly(A) tail formation and length control prompted us to examine possible roles for this protein in SOX-induced hyperadenylation and mRNA turnover as well. To this end, we monitored SOX activity by northern blotting and quantitative real-time PCR (qPCR) upon siRNA-mediated knockdown of either PABPC or PABPN. Indeed, northern blotting showed there was a significant decrease in the ability of SOX to promote GFP mRNA degradation upon knockdown of either PABPN or PABPC (Figure 5A and 5B). In contrast, SOX-expressing cells transfected with a control nonspecific siRNA showed robust turnover of the GFP reporter mRNA. These effects are highly specific; we have performed siRNA-mediated knockdowns of approximately 10 other cellular proteins involved in mRNA stability with no resulting decrease in SOX function (Figure S4, unpublished data). Additionally, the siRNA treatment did not affect the levels of SOX protein expression or the mRNA levels of the GFP reporter in the absence of SOX (Figure 5A and 5B). These results were confirmed by qPCR analysis of GFP mRNA levels from these samples, which showed a strong inhibition of SOX host shutoff activity upon PABPN or PABPC knockdown (Figure 5C). Of note, we have consistently observed that knockdown of PABPN, but not PABPC, blocks hyperadenylation detected by northern blotting (compare lanes 3 and 4 in Figure 5B). In addition, we monitored hyperadenylation of endogenous messages in SOX-expressing cells upon PABPC knockdown using oligo(dT) in situ hybridization (Figure S5). In agreement with our northern blots, siRNA-mediated depletion of PABPC also failed to inhibit SOX-induced hyperadenylation in these experiments. Although similar experiments were also performed upon PABPN siRNA treatment, these were more difficult to interpret because a fraction of the vector-transfected control cells exhibited enhanced nuclear dT staining (possibly due to mRNA export defects). However, it appeared as though a reduced number of SOX-expressing cells lacking PABPN exhibited hyperadenylation (Figure S5). Collectively, these data suggest that hyperadenylation may be necessary, but not sufficient, for SOX-mediated RNA turnover, and that the contributions of PABPC to SOX function may be downstream of those of PABPN. That PABPC is required for the mRNA turnover activity of SOX suggests its host shutoff-dependent nuclear import is not simply a byproduct of hyperadenylation, but rather plays an integral role in directing turnover of cellular transcripts in the presence of SOX. Indeed, siRNA-induced knockdown of PAPII (which inhibits hyperadenylation; see Figure 2) produces no defect in SOX-induced PABPC relocalization (Figure S6), indicating that hyperadenylation is not a prerequisite for nuclear import of PABPC.

### Role for the Poly(A) Tail in Facilitating mRNA Degradation by SOX

**Figure 6:**
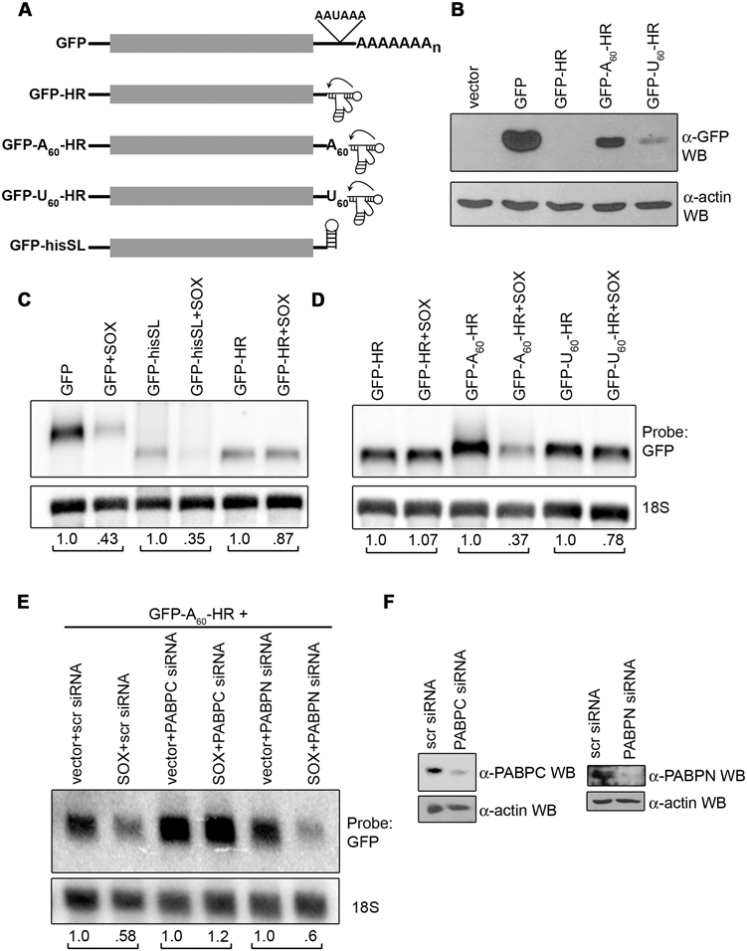
Direct contribution of a poly(A) tail towards mRNA destruction by SOX. (A) Diagram of reporter constructs either containing the WT polyadenylation signal sequence (GFP) or lacking the polyadenylation signal and terminating in either a hammerhead ribozyme element (GFP-HR) with or without a preceding templated 60-nt poly(A) or poly(U) tail (GFP-A_60_-HR and GFP-U_60_-HR, respectively), or the histone 3′ SL (GFP-hisSL). (B) A total of 200 ng of each GFP plasmid was transfected into HEK 293T cells, which were harvested 24 h later and subjected to western blotting with GFP antibodies to show relative protein expression from each construct. (C and D) HEK 293T cells were transfected with the indicated GFP construct alone or together with a SOX expression construct at a 1∶2 ratio (50 ng of GFP, 100 ng of SOX). Total RNA was harvested from each sample 24 h post-transfection and northern blotted with GFP and 18S probes. (E and F) HEK 293T cells were transfected twice sequentially with the indicated duplex siRNA oligos. Twenty-four hours after the final siRNA transfection, the cells were transfected with the GFP-A_60_-HR plasmid alone or together with a SOX expression plasmid at a 1∶2 ratio, and incubated in media containing 5 ng/ml LMB for 6 h prior to harvesting. Twenty-four hours later, the cells were harvested either for RNA and northern blotted with GFP and 18S probes (E), or for protein and western blotted with PABPC, PABPN, and actin (loading control) antibodies to monitor the efficiency of siRNA-induced knockdown (F). Quantification (normalized to 18S levels) is shown below each northern blot. The level of each GFP mRNA in the absence of SOX was set to 1.0, and the corresponding level of that particular mRNA in the presence of SOX was then calculated.

Our data indicate that polyadenylation plays a key role in the host shutoff function of SOX. We therefore sought to more directly evaluate the contribution of a poly(A) tail towards SOX-induced mRNA turnover by preventing polyadenylation of the GFP reporter message. This was accomplished by replacing the portion of the GFP 3′ UTR containing the AAUAAA polyadenylation signal sequence with a self-cleaving hammerhead ribozyme element (GFP-HR; Figure 6A). The 3′ end cleavage of this mRNA is mediated by the ribozyme rather than cellular machinery, and it is not polyadenylated at steady state and should not associate with the PABPs. Notably, although SOX promoted turnover of the polyadenylated GFP message, it failed to degrade the GFP-HR RNA (Figure 6C). To determine whether absence of polyadenylation was the primary cause for the inability of SOX to promote GFP-HR mRNA turnover, we next generated GFP constructs lacking the polyadenylation signal sequence but containing a templated stretch of either 60 adenosine residues (GFP-A_60_-HR) or, as a control, 60 uridine residues (GFP-U_60_-HR) immediately upstream of the ribozyme cleavage site (Figure 6A). The minimum poly(A) interaction site size for PABPN is 10 nt [35] and for PABPC is 12 nt [36], although when coated along a poly(A) tail, each PABPC protein covers approximately 25 nt [38]. Thus, the 60-nt templated poly(A) tail is of sufficient length to bind multiple copies of PABPC and/or PABPN. As would be predicted, the GFP-HR mRNA fails to be translated, whereas addition of the A_60_ tail partially rescues this defect, and very weak protein expression is observed with a U_60_ tail (Figure 6B). Significantly, the presence of a templated poly(A) tail, but not a poly(U) tail, was sufficient to reinstate SOX-induced degradation of the GFP reporter (Figure 6D), indicating that a poly(A) tail specifically participates in targeting mRNAs for turnover by SOX. We observed similar results with the SOX homolog from a related γ-herpesvirus, MHV68 (Figure S7).

To examine the requirement for PABPC and PABPN in SOX-induced turnover of the GFP-A_60_-HR mRNA, we performed siRNA-mediated knockdowns of these factors and monitored the resulting ability of SOX to degrade the GFP message (Figure 6E and 6F). The cells were also treated with LMB to determine whether SOX-induced hyperadenylation occurs on this ribozyme-terminated mRNA. Cells transfected with a scramble siRNA showed SOX-induced turnover of the GFP-A_60_-HR transcript, but no hyperadenylation (Figure 6E), as anticipated given our observation that hyperadenylation requires the machinery involved in cellular 3′ end formation, which does not participate in processing of the HR constructs. Knockdown of PABPN, which is required for poly(A) tail formation and hyperadenylation, did not prevent SOX-mediated turnover of GFP-A_60_-HR. However, knockdown of PABPC effectively prevented SOX-mediated turnover of this mRNA. Thus, PABPN is necessary for SOX-induced hyperadenylation and destruction of mRNAs processed by the cellular 3′ end machinery but is not required if a poly(A) tail is already in place, whereas PABPC is a critical cofactor for SOX-induced destruction of already polyadenylated mRNAs. Reproducibility of all northern blotting results shown in Figure 6 was demonstrated by quantification of multiple replicates (*n*≥3; Figure S8).

Finally, histone mRNAs are the only known cellular mRNAs lacking poly(A) tails, as they instead terminate in a 3′ stem loop (SL) structure that recruits a number of the same processing and degradation factors as poly(A) mRNAs [38],[39]. We examined whether a GFP construct containing the histone SL and the downstream element required for termination would be sensitive to SOX-mediated turnover (Figures 6C and S7). Interestingly, histone SL-terminating mRNA was degraded by SOX, suggesting that this unique message may recruit one or more factors in a poly(A)-independent manner that facilitate SOX targeting. This is in contrast to other non-polyadenylated RNAs (e.g., ribosomal RNAs), which are not subject to SOX-mediated degradation [5]. An important future direction will be to define the specific elements or factors that render RNAs like the histone mRNA susceptible to SOX-mediated RNA turnover, as they are anticipated to identify areas of convergence between polyadenylation-dependent and -independent pathways of mRNA degradation.

## Discussion

### A Novel Mechanism of Virus-Induced Host Shutoff

The ability to regulate cellular gene expression is a key aspect of the lifecycles of a diverse array of viruses. Global inhibition of cellular protein synthesis serves not only to ensure maximal viral gene expression by diverting the cellular resources towards the virus, but also assists in evasion of host immune responses detrimental to viral replicative success. Although the outcome of host shutoff may be similar for some pathogens, the mechanisms they use to achieve this endpoint are quite distinct. For example, poliovirus prevents cap-dependent translation by cleavage of eIF4G and PABP [40],[41], vesicular stomatitis virus blocks nuclear mRNA export via disruption of Rae1 function [42], and herpes simplex virus (HSV) both inhibits splicing and encodes a ribonuclease that degrades cytoplasmic mRNA [43]–[46]. Although KSHV infection elicits global mRNA turnover via the activity of SOX, the mechanisms driving this phenotype remained enigmatic.

**Figure 7:**
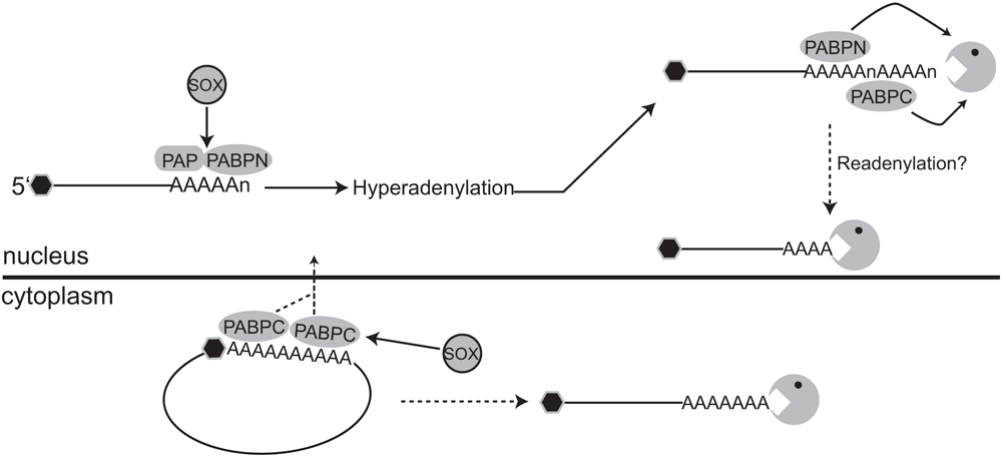
Proposed model for SOX-induced hyperadenylation and host shutoff activity. SOX stimulates mRNA hyperadenylation in a manner dependent on the activity of both PAPII and PABPN. This aberrant 3′ processing event may trigger destruction of these mRNAs by quality control ribonucleases (shown as a pacman), possibly recruited to the mRNAs by PABPN and the nuclear relocalized PABPC. Within the cytoplasm, removal of PABPC could also be envisioned to negatively affect the stability of mRNAs by rendering their 3′ termini unprotected from ribonuclease digestion and decreasing translation efficiency.

Here, we demonstrate that SOX engages in a novel mechanism of host shutoff involving aberrant mRNA polyadenylation (Figure 7). To our knowledge, this is the first example of enhanced RNA turnover coupled to hyperadenylation by PAPII in metazoans. We further show that degradation of these cellular transcripts requires nuclear and cytoplasmic PABPs, the latter of which undergoes striking nuclear relocalization during KSHV infection. Manipulation of this cellular RNA 3′ processing event and PABPC nuclear recruitment require the host shutoff activity of SOX, and are therefore intimately linked to KSHV-induced transcriptome turnover. Although we do not yet know the identity of the ribonuclease ultimately responsible for mRNA destruction in the presence of SOX, we propose that SOX-induced alterations in mRNA processing events may render these nascent RNAs targets of cellular quality control machinery.

### Links between Polyadenylation and mRNA Turnover

Several lines of evidence suggest that polyadenylation plays an integral role in SOX-mediated mRNA turnover. First, SOX mutants selectively defective for mRNA turnover, but retaining the conserved DNase activity, fail to promote hyperadenylation. Second, siRNA-mediated knockdown of PABPN, which stimulates poly(A) polymerase activity and has important roles in poly(A) tail formation and length control [16], reduces SOX-induced hyperadenylation and mRNA turnover. Finally, ribozyme-terminating transcripts that bypass cellular 3′ end cleavage and polyadenylation cannot be targeted by SOX for destruction, whereas turnover is reinstated upon addition of a templated poly(A) but not poly(U) tail. Collectively, these data suggest that the poly(A) tail serves as a signal for degradation in SOX-expressing cells and/or participates in the recruitment of decay factors, perhaps via the PABPs (Figure 7).

Polyadenylation-triggered mRNA decay is well established in prokaryotes such as *Escherichia coli* [47]. Both mature and fragmented bacterial mRNAs can be polyadenylated, but rather than stabilizing the messages (as generally occurs in eukaryotes), here polyadenylation facilitates RNA degradation [19]. In this regard, prokaryotic poly(A) tails are thought to serve as unstructured “landing pads” for exoribonucleases, thereby assisting their progression through structured regions of the RNA. In contrast, the eukaryotic nuclear mRNA polyadenylation reaction that is coupled to 3′ end cleavage and processing generally helps protect messages from exonucleolytic attack, and mutation of poly(A) polymerase results in rapid depletion of mRNAs [48],[49]. However, it is likely that polyadenylation can also serve as an important signaling mechanism for the cell to monitor the fidelity of RNA processing in eukaryotes. Compelling evidence linking polyadenylation directly to destruction of eukaryotic RNAs emerged in yeast with the discovery of the TRAMP nuclear polyadenylation complex that tags aberrant messages with short poly(A) tails to stimulate turnover [20]–[22]. Indeed, accumulation of polyadenylated forms of RNAs that do not normally have poly(A) tails, such as small nucleolar RNA (snoRNA) [50],[51], ribosomal RNA (rRNA) precursors [52]–[54], and intergenic transcriptional events [22],[55] can be detected in yeast lacking the exosome component Rrp6; these are presumably TRAMP-labeled degradation intermediates stabilized in the absence of surveillance-mediated decay. Although a number of homologs of the TRAMP polymerase Trf4 exist in humans, none have yet been shown to function in an analogous manner. However, short poly(A) tails reminiscent of TRAMP products have been detected on cotranscriptionally cleaved β-globin pre-mRNAs in mammalian cells [28], as well as on human mitochondrial transcripts and rRNAs [26],[27].

Proper 3′ end formation and polyadenylation are required for mRNA export into the cytoplasm, and defects in these processes trigger nuclear retention and RNA destruction by quality control pathways [56]. Interestingly, yeast nuclear export and 3′ end processing mutants can lead to hyperadenylated transcripts that accumulate at the site of transcription [23]–[25]. We therefore predict that hyperadenylated messages produced in SOX-expressing cells would be regarded as aberrantly processed and retained in the nucleus for eventual destruction. Of note, we have not observed significant defects in SOX activity upon depletion of the human exosome components (Figure S4), suggesting that this quality control complex does not play a major role in SOX-mediated host shutoff. This is perhaps not unexpected, as exosome depletion would be anticipated to only affect turnover of the nascent hyperadenylated nuclear transcripts, rather than the bulk of transcripts in the cytoplasm that become destabilized coincident with PABPC import. This idea is further supported by our observation that inhibiting hyperadenylation by depleting PAPII results in partial reduction rather than complete inhibition of SOX-induced mRNA turnover. It should be noted, however, that the nuclear exosome is apparently more refractory to siRNA-mediated turnover than the cytoplasmic exosome [57]. It is therefore possible that in our knockdown experiments, enough exosomal proteins remain in the nuclear fraction to promote turnover of the hyperadenylated messages. An alternative possibility is that other enzymes are involved in polyadenylation-triggered nuclear mRNA turnover during KSHV infection.

Given our evidence linking polyadenylation to host shutoff, the degradation of a reporter bearing the histone mRNA termination signals by SOX was unexpected. We envision at least two possible scenarios explaining degradation of this non-polyadenylated transcript. First, despite lacking the canonical mRNA cleavage and termination signals or a poly(A) tail, the histone mRNA 3′ end is nonetheless able to recruit a significant number of the same factors involved in nuclear RNA processing as well as cytoplasmic RNA turnover as polyadenylated mRNAs [39],[58],[59]. The histone mRNA 3′ end SL structure may therefore be able to bind proteins normally associated with a poly(A) tail that are necessary for SOX targeting. PABPC, for example, has been shown to associate with a non-poly(A) element within the MKK-2 mRNA 3′ UTR to control its stability [60], although to our knowledge, there is currently no evidence that it binds histone mRNA. Secondly, it is formally possible that in contrast to uninfected cells, upon SOX expression, this mRNA becomes polyadenylated and thus subject to destruction. An important future goal will be to determine whether either of these possibilities is correct.

### Implications for the Relocalization of Poly(A) Binding Protein

Whereas PABPC is known to shuttle between the nucleus and cytoplasm, its steady-state localization is cytoplasmic, and possible roles for it in the nucleus remain largely unknown. Recent observations, however, suggest that PABPC is likely to have functions in the nucleus, because it interacts with polyadenylated nuclear pre-mRNAs as well as with PAP, suggesting that its association with the pre-mRNAs occurs during tail formation in the nucleus [61]. However, our observation that siRNA-mediated knockdown of PABPC prevents mRNA turnover, but not hyperadenylation by SOX, indicates that PABPC functions downstream of PABPN, perhaps in the recruitment of RNA decay factors. The fact that PABPC but not PABPN depletion blocks SOX-mediated degradation of the ribozyme-terminating GFP with a 60-nt templated poly(A) tail further bolsters this conclusion.

It should be noted that four cytoplasmic PABPs have been identified in human cells [62]. In addition to PABPC1, HEK 293T cells also likely express PABPC4 (iPAPB) [63] and PABPC5 (X-linked PABP) [64], whereas PABPC3 is reported to be testes specific [65]. Our knockdowns selectively targeted PABPC1, thought to be the predominant PABPC in these cells, and removal of this protein clearly has detrimental effects on SOX activity. However, there is extensive sequence homology between the PABPCs [62], and the PABPC1 antibody is predicted to cross-react with at least PABPC4 as well; it is therefore possible that SOX induces nuclear relocalization of multiple lineages of PABPC during host shutoff. We hypothesize that the presence of these additional PABPCs is what prevents general mRNA destabilization upon siRNA-mediated depletion of PABPC1 in the absence of SOX. In this regard, an additional consideration is that while PABPC1 depletion did not abrogate the hyperadenylation phenotype, it remains to be determined whether other PABPC proteins are similarly dispensable for this function.

Surveillance-mediated destruction of aberrantly processed pre-mRNAs could explain how cellular messages are depleted from the nucleus during lytic KSHV infection, but what about the abundance of cytoplasmic cellular transcripts? Given that the average half-lives of mammalian mRNAs are relatively long [66] and we have shown that SOX-expressing cells exhibit enhanced turnover of cytoplasmic messages, we predict that SOX activity must stimulate both nuclear and cytoplasmic mRNA decay either by distinct or overlapping mechanisms. In this regard, it is significant that PABPC has established roles in preserving cytoplasmic mRNA integrity by promoting mRNA circularization via interactions with eIF4G to enhance stability and translation via the closed loop model [16]. Thus, removal of PABPC from the cytoplasm could render these messages less translatable, unprotected at their 3′ termini, and more susceptible to nucleolytic attack, in agreement with our observation that the cytoplasmic GFP mRNA half-life is reduced in SOX-expressing cells. Indeed, multiple RNA viruses with unique translational strategies have evolved means to disrupt PABPC activity presumably to facilitate selective translation of viral messages and promote host translational shutoff; enteroviruses, caliciviruses, and HIV encode proteases that cleave PABPC [67]–[69], and the rotavirus NSP3 protein competes with PABPC for eIF4G binding and promotes PABPC relocalization [70]–[72]. Thus, multiple diverse groups of viruses have all evolved strategies to target this cellular factor, presumably to divert resources away from cellular gene expression. We hypothesize that SOX host shutoff activity consists of a nucleus-specific component requiring PABPC and PABPN-stimulated aberrant mRNA hyperadenylation and turnover, and a cytoplasmic component involving inhibition of mRNA translation followed by destabilization coincident with PABPC depletion. KSHV may have additional roles for PABPC during infection, as a recent report showed a limited amount of the K10/K10.1 viral protein associates with PABPC in the nucleus during the lytic cycle, although the functional significance of this observation remains unknown [73].

### Models for Viral mRNA Escape from Turnover

Finally, one particularly intriguing issue is how viral messages manage to evade host shutoff. KSHV mRNAs closely resemble cellular transcripts, in that they are 5′ capped and polyadenylated, some are spliced, and they are transcribed, processed, and translated using cellular machinery; yet these messages must escape the mRNA destruction fate suffered by cellular transcripts. Our findings suggest that successful viral gene expression during host shutoff requires navigating at least two obstacles: first, evading aberrant 3′ end mRNA processing and destruction in the nucleus and, second, keeping the messages stable and efficiently translated in the cytoplasm in the face of significantly reduced PABPC levels. One clever mechanism of evading nuclear degradation has been delineated for the highly abundant KSHV noncoding PAN RNA; this polyadenylated nuclear transcript contains a 79-nt RNA element (termed the ENE) near its 3′ end which acts post-transcriptionally to stabilize and enhance the nuclear levels of PAN or other reporter RNAs [74]. Modeling experiments predict the ENE folds into a secondary structure reminiscent of box H/ACA snoRNAs, and indeed, this element can form intermolecular interactions with the PAN poly(A) tail in a manner that blocks deadenylation, thereby stabilizing the RNA [75]. An important future challenge will be to delineate the mechanisms by which the bulk of the remaining viral mRNAs achieve these tasks, as such information may provide clues as to how cellular quality control checkpoints could be bypassed during viral infection or other human disease.

## Materials and Methods

### Plasmids

GFP-HR was created by replacing the AAUAAA signal sequence of pd2EGFP-N1 (Clontech) with a hammerhead ribozyme (CCTGTCACCGGATGTGTTTTCCGGTCTGATGAGTCCGTGAGGACGAAACAGG) by deleting the NotI/AflII-flanked poly-A signal and cloning in annealed hammerhead ribozyme oligos with NotI and AflII overhangs. GFP-A_60_-HR and GFP-U_60_-HR were generated by ligation of an A_60_ oligo into the Not1 site of GFP-HR in the forward or reverse orientation, respectively. GFP-hisSL was created by replacing the AAUAAA signal sequence of pd2EGFP-N1 (Clontech) with a histone stem and downstream element (HDE) sequence (ATGTAAGTCTAGAGGATGGGGAGCAAAAGGCTCTTTTCAGAGCCACCCACTGAATCAGATAAAGAGTTGTGTCACGGTAGCCA) by deleting the NotI/AflII flanked poly-A signal and cloning in annealed histone stem and downstream element oligos with NotI/AflII overhangs. The cloning of pCDEF3-SOX, pCDEF3-HA-HSV AE [5], and the pCDEF3-SOX single-function mutants (Q129H, T241, and P176S) [4] were described previously. Mutant L20/23A was generated by overlapping PCR and then cloned into the EcoR1/Not1 sites of pCDEF3.

### Cells and Infections

HEK 293T cells (American Type Culture Collection) were maintained in DMEM supplemented with 10% FBS. Telomerase-immortalized microvascular endothelial (TIME) cells [76] were maintained using EBM-2 medium bullet kits (Clonetics). TIME cells were infected with KSHV and lytically reactivated with an adenoviral vector expressing the lytic switch protein RTA as described previously [77].

### siRNA Knockdown

siRNA duplex oligos (Bioneer; Dharmacon) were generated against the following target sequences: PAPII (accession number NM_032632) siRNA #1: CTGCGTACTTACACAGAAA, PAPII siRNA #2: GATTAGGAGTGCATACAAA; PAPγ (GenBank accession number NM_022894) siRNA #1: CAACAGAATTCTACGTATA, PAPγ siRNA #2: GGAGAAACAGAAAGGAATA; PABPC1 (GenBank accession number NM_002568) siRNA #1: GAAAGGAGCTCAATGGAAA; PABPC1 siRNA #2: GGACAAATCCATTGATAAT; and PABPN (GenBank accession number NM_004643) siRNA #1: GTAGAGAAGCAGATGAATA; PABPN siRNA #2: CTATTTAGAGGAAGGCAAA. Nonspecific control siRNA duplexes #1 and #2 were purchased from Ambion. HEK 293T cells were transfected with siRNA oligos at a final concentration of 200 nM using Lipofectamine 2000 (Invitrogen), both at 48 h and 24 h prior to DNA transfections, and harvested 24 h after the DNA transfections for total RNA and protein or processed for in situ hybridization.

### In Situ Hybridization, Immunofluorescence Assays

Cells were harvested 24 h post DNA transfection for both oligo(dT) in situ hybridization and immunofluorescence analyses. In situ samples were processed as described (http://www.singerlab.org/protocols) using 2 ng/µL of AlexaFluor 546-labeled oligo-dT(_15_) (Molecular Probes). After oligo hybridization, samples were incubated with either α-SOX J5803 or α-HA (Abcam) primary antibodies at a 1∶500 dilution in 2× SSC, 0.1% triton X-100 for 3 h at 37°C, then subsequently with Alexa fluor 488-labeled goat α-rabbit secondary antibodies (Molecular Probes) and mounted with DAPI-containing Vectashield mounting medium (Vector Labs). IFA not performed in conjunction with in situ hybridization was done as described previously [77] using either SOX J5803 polyclonal antibodies (1∶500 dilution), 10E10 monoclonal PABPC antibodies (generously provided by Dr. G. Dreyfuss) (1∶1,000 dilution), rabbit polyclonal PABPC #39473 antibodies (generously provided by Dr. R. Andino) (1∶100 dilution), rabbit polyclonal PABPN antibodies (generously provided by Dr. E. Wahle) (1∶250 dilution), monoclonal HA 12CA5 antibodies (Abcam) (1∶500 dilution), and Alexa Fluor 488- or 546-labeled goat α-rabbit or α-mouse secondary antibodies (1∶1,500 dilution) (Molecular Probes).

### Cell Extracts, Immunoblots, Northern Blots

Lysates were prepared in RIPA buffer (50 mM Tris-HCl [pH 8.0], 150 mM Nacl, 1% [v/v] Nonidet P-40, 0.5% [w/v] sodium deoxycholate, 0.1% [w/v] sodium dodecyl sulfate [SDS]) containing protease inhibitors (Roche). Equivalent amounts of each sample were subjected to immunoblotting with the following antibodies: PAPII (1∶1,000 dilution) (generously provided by Dr. J. Manley), PAPγ (1∶1,000 dilution) (generously provided by Dr. A. Virtanen), HA (Abcam) (1∶5,000 dilution), PABPC #39473 (1∶2,500), or SOX J5803 (1∶5,000 dilution) (see below), and either HRP-conjugated goat-α-rabbit or goat-α-mouse secondary antibodies (Southern Biotechnology Assoc.). Rabbit polyclonal antisera were raised against a maltose binding protein (MBP)-tagged full-length SOX by standard methods [78].

Where indicated, the transfected HEK 293T cells were treated with 5 ng/ml leptomycin B (LMB) (Sigma) for 6–12 h prior to RNA isolation. Total cellular RNA was isolated using RNA-BEE (Tel-Test), resolved on 1.2% agarose-formaldehyde gels, and probed with a ^32^P-labeled GFP DNA probe generated using the RediPrime II random prime labeling kit (Amersham). Membranes were subsequently incubated with an 18S probe as a loading control. For half-life analysis, cells were treated with LMB for 12 h, then washed with PBS and transferred to medium containing 1 µg/ml Actinomycin D (ActD) minus LMB for the indicated times. For fractionation studies, NE-PER Nuclear and Cytoplasmic Extraction Reagents Kit (Pierce) or Paris Kit (Ambion) was used according to the manufacturer's instructions. The level of GFP mRNA was divided by the corresponding level of 18S rRNA to correct for errors in sample loading. The log of normalized data was then plotted versus the time of treatment of ActD. The reported data are the means of a minimum of three independent experiments. Northern blots were analyzed using a Typhoon 8600 phosphorimager (Molecular Dynamics).

RNaseH digestions were performed by combining 10 µg of RNA with 500 pmol of oligo(dT) primer in a 25 µl reaction, incubating at 65°C for 8 min, then adding 1 U of RNaseH (New England Biolabs), RNaseH buffer to 1×, and 40 U of RNasin (Promega). Reactions were incubated at 37°C for 30 min, then terminated by adding 1 µl of 0.5 M EDTA (pH 8.0) and ethanol precipitating the RNA prior to gel electrophoresis.

### Quantitative Real-Time PCR (qPCR)

cDNAs were synthesized from 1 µg of total RNA using AMV reverse transcriptase (Promega), diluted 1∶5, and used directly for qPCR analysis. GFP cDNA was amplified using the 5′ primer 5′CAACAGCCACAACGTCTATATCATG and 3′ primer 5′ATGTTGTGGCGGATCTTGAAG, along with a Taqman probe 5′FAM-CAAGCAGAAGAACGGCATCAAGGTGA-BHQ1. Taqman Ribosomal RNA Control Reagent (Applied Biosystems) with VIC-labeled probe and forward and reverse primers for human 18S rRNA was used as a loading control. Standard curves were prepared for each primer/probe set using 10-fold serial dilutions of either the 97-nt GFP fragment or the 55-nt 18S fragment derived from a pGem-T-easy vector (Promega). The qPCR reaction was performed using Taqman Gene Expression Mix (Applied Biosystems) in the presence of 100 nM GFP primers, 200 nM GFP probe, 50 nM 18S rRNA primers, 200 nM 18S rRNA probe, and 9 mM MgCl_2_. The level of GFP mRNA was calculated using a mathematical model of relative expression in qPCR [79] to quantify the relative level of GFP mRNA in comparison to the 18S rRNA.

## Supporting Information

Figure S1 Leptomycin B treatment significantly increases the amount of SOX protein in the nucleus. HEK 293T cells were transfected with a plasmid expressing SOX and, 24 h post-transfection, either left untreated or treated with 5 ng/ml of leptomycin B (LMB) for 6 h. SOX expression and localization was then monitored by immunofluorescence analysis using SOX polyclonal antibodies.
(0.46 MB TIF)

Figure S2 Quantification of poly(A) RNA accumulation via PAPII in SOX-expressing cells. HEK 293T cells were either mock transfected or transfected twice with PAPII or PAPγ duplex siRNA oligos or nonspecific control siRNA oligos (scramble si). Twenty-four hours after the final siRNA transfection, the cells were transfected with a DNA plasmid expressing the GFP reporter alone or together with SOX and, 24 h later, harvested for RNA and northern blotted with GFP and 18S probes. GFP mRNA levels were normalized to 18S rRNA. The level of each GFP mRNA in the absence of SOX was set to 1.0, and the corresponding level of that particular mRNA in the presence of SOX was then calculated upon PAPII or PAPγ knockdown. The data are the mean±the standard error between experimental replicates (*n* = 3).
(0.17 MB TIF)

Figure S3 Gels showing half-life measurements of GFP mRNA in the cytoplasm of HEK 293T cells either in the absence or presence of SOX. HEK 293T cells were transfected with GFP alone (100 ng) or together with a SOX expression plasmid (200 ng). Twenty-four hours post-transfection, cells were treated with 1 µg/ml actinomycin D for the indicated time to halt transcription. Cytoplasmic RNA was then extracted and northern blotted with GFP and 18S probes.
(8.45 MB TIF)

Figure S4 The exosome does not play an essential role in SOX-induced mRNA destruction. HEK 293T cells were transfected with hRrp41 or PM/Scl-100 (hRrp6) duplex siRNA oligos or a combination of hRrp41+PM/Scl-100 oligos, or a nonspecific control siRNA oligo (scr si). Twenty-four hours after the siRNA transfection, the cells were transfected with the indicated DNA plasmid(s) expressing GFP±SOX; each sample was split in half and, 72 h later, either harvested for protein and immunoblotted with polyclonal hRrp41 or PM/Scl-100 antibodies to measure the efficiency of siRNA-mediated knockdown (A), or harvested for total RNA and subjected to quantitative real-time PCR using GFP specific primers (B). As reported previously, Rrp41 knockdown leads to destabilization of other exosome components including PM/Scl-100 [57]. The asterisk in (A) marks the location of the PM/Scl-100 protein. Lines through gels indicate where an intervening lane was cropped out of the image.
(0.97 MB TIF)

Figure S5 Oligo(dT) in situ hybridization in SOX-expressing cells following PABPC or PABPN knockdown. HEK 293T cells were transfected twice sequentially with either scramble control duplex siRNA oligos, or PABPC or PABPN duplex siRNA oligos. Twenty-four hours after the last siRNA transfection, the cells were split 1∶2 and transfected with empty vector or a SOX expression plasmid. Twenty-four hours later, half the samples were harvested for protein and western blotted for PABPC and PABPN to gauge the level of knockdown, as well as PAPII (as a loading control) and SOX (A). The other half of the samples were subjected to oligo(dT) in situ hybridization (left) and IFA with SOX antibodies (center) to monitor the ability of SOX to promote hyperadenylation of endogenous mRNAs in the absence of PABPC or PABPN (B). The right panels represent a merge of the oligo dT and SOX images.
(4.19 MB TIF)

Figure S6 SOX-induced nuclear import of PABPC is not an indirect result of hyperadenylation. HEK 293T cells were transfected twice sequentially with either scramble control duplex siRNA oligos or PAPII siRNA oligos (to block hyperadenylation). Twenty-four hours after the last siRNA transfection, the cells were split 1∶2 and transfected with empty vector or a SOX expression plasmid. Twenty-four hours later, half the samples were harvested for protein and western blotted for PAPII to gauge the level of knockdown, as well as PABPC (as a loading control) and SOX (A). The other half of the samples were subjected to immunofluorescence analysis with PABPC (left) and SOX (center) antibodies to monitor the ability of SOX to promote import of endogenous PABPC in the absence of hyperadenylation (B). The right panels represent a merge of the PABPC and SOX images.
(4.63 MB TIF)

Figure S7 Degradation of GFP-HR and GFP-hisSL mRNAs by KSHV and MHV68 SOX. (A and B) HEK 293T cells were transfected with each of the indicated GFP plasmids alone or together with KSHV SOX (A) or the MHV68 SOX homolog (mSOX [B]) at a 1∶2 ratio. Total RNA was harvested from each sample 24 h post-transfection and northern blotted with GFP and 18S probes. (C) HEK 293T cells were transfected with the indicated plasmid (WT GFP or GFP terminating with the histone SL and termination sequences (GFP-hisSL); 24 h later, protein was harvested and western blotted with GFP antibodies to compare the level of protein expression from the GFP-hisSL construct relative to WT GFP.
(0.48 MB TIF)

Figure S8 Quantification of SOX-induced mRNA turnover shown in Figure 6. (A and B) Quantification of SOX-induced turnover of GFP mRNA in the presence or absence of a poly(A) tail. HEK 293T cells were transfected with the indicated wild-type or ribozyme-terminating GFP construct in the presence or absence of SOX. Total RNA was harvested 24 h post-transfection and northern blotted with GFP and 18S probes. The level of each GFP or GFP-HR mRNA in the absence of SOX was set to 1.0 after normalization to 18S rRNA, and the corresponding level of that particular mRNA in the presence of SOX was then calculated. The data are the means±the standard error between experimental replicates (*n* = 4 for [A], *n* = 3 for [B]). (C) Quantification of the contribution of PABPC and PABPN towards GFP-A_60_-HR mRNA destruction by SOX. HEK 293T cells were transfected twice sequentially with PABPC or PABPN duplex siRNA oligos or nonspecific control siRNA oligos (scramble si). Twenty-four hours after the final siRNA transfection, the cells were transfected with the GFP-A_60_-HR plasmid alone or together with a SOX expression plasmid. Twenty-four hours later, the cells were harvested and subjected to northern blot analysis. Quantification was performed as described above (*n* = 3).
(0.25 MB TIF)

## Abbreviations

KSHV: Kaposi's sarcoma-associated herpesvirus
LMB: leptomycin B
nt: nucleotides
qPCR: quantitative real-time polymerase chain reaction
rRNA: ribosomal RNA
siRNA: small interfering RNA
SL: stem loop
WT: wild-type
