# Supplementary figures and images for: Aberrant Herpesvirus-Induced Polyadenylation Correlates With Cellular Messenger RNA Destruction
Source: PLoS Biol. 2009 May 26;7(5):e1000107. doi: 10.1371/journal.pbio.1000107 (PMC2680333; doi:10.1371/journal.pbio.1000107)

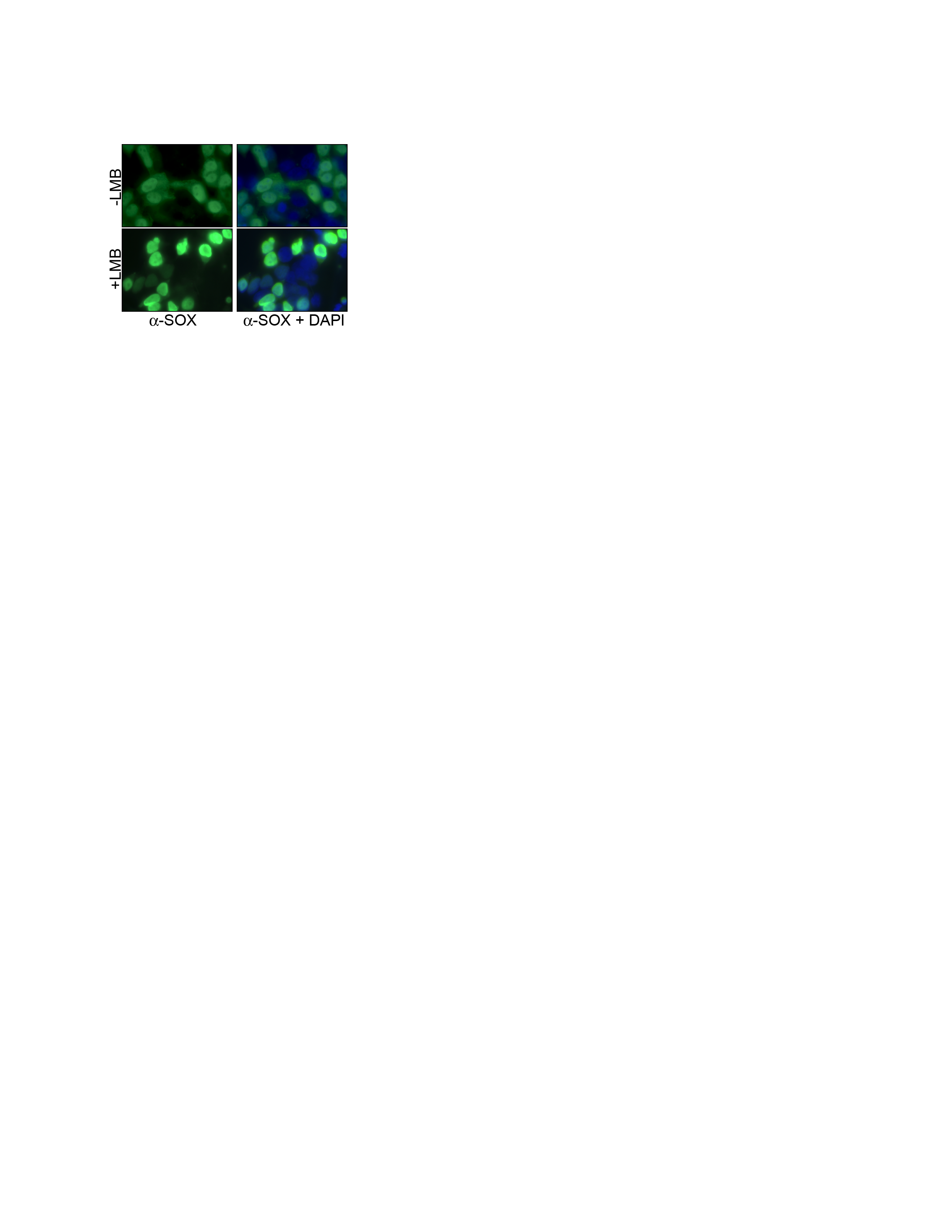

Supplement: Figure S1 — Leptomycin B treatment significantly increases the amount of SOX protein in the nucleus. HEK 293T cells were transfected with a plasmid expressing SOX and, 24 h post-transfection, either left untreated or treated with 5 ng/ml of leptomycin B (LMB) for 6 h. SOX expression and localization was then monitored by immunofluorescence analysis using SOX polyclonal antibodies. (0.46 MB TIF) [file pbio.1000107.s001.tif]

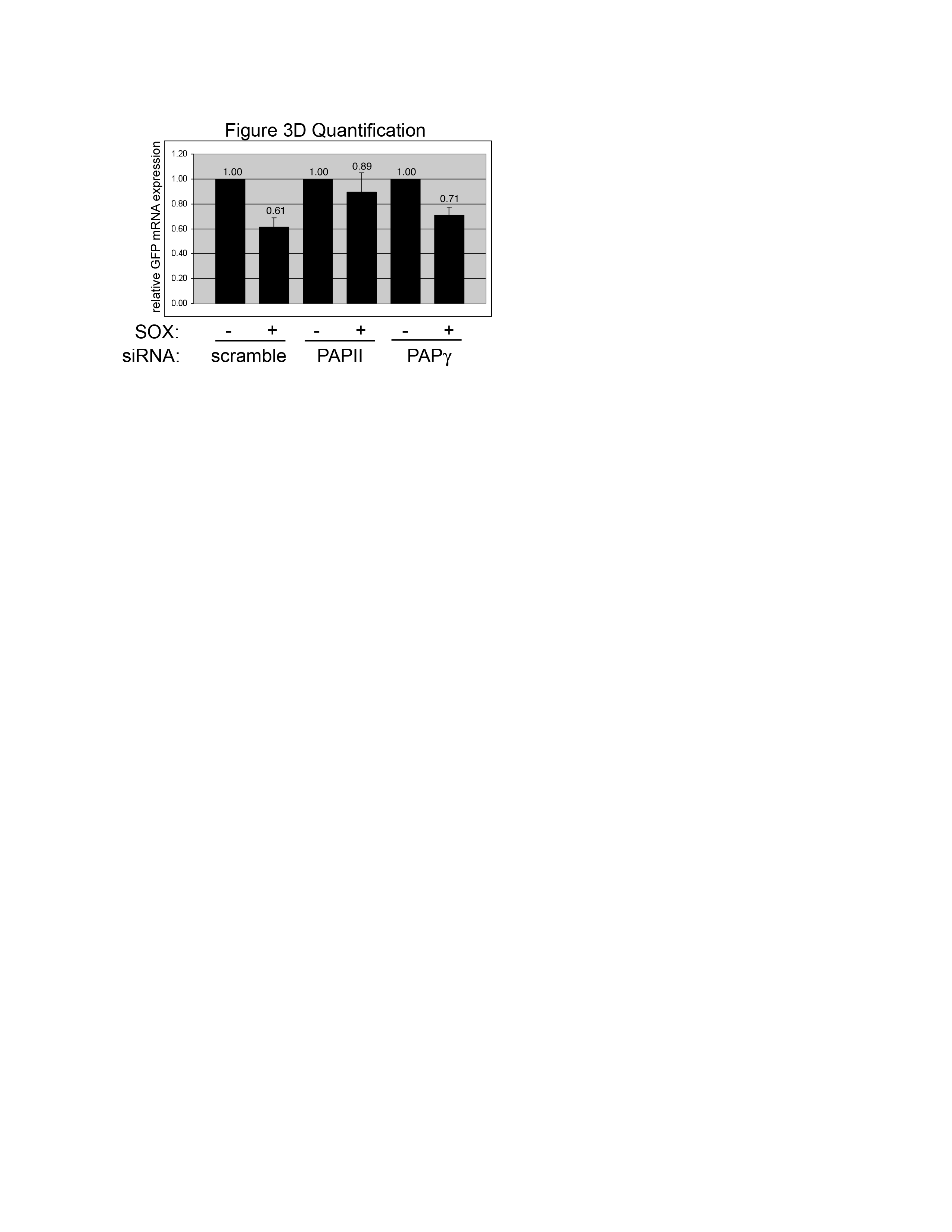

Supplement: Figure S2 — Quantification of poly(A) RNA accumulation via PAPII in SOX-expressing cells. HEK 293T cells were either mock transfected or transfected twice with PAPII or PAPγ duplex siRNA oligos or nonspecific control siRNA oligos (scramble si). Twenty-four hours after the final siRNA transfection, the cells were transfected with a DNA plasmid expressing the GFP reporter alone or together with SOX and, 24 h later, harvested for RNA and northern blotted with GFP and 18S probes. GFP mRNA levels were normalized to 18S rRNA. The level of each GFP mRNA in the absence of SOX was set to 1.0, and the corresponding level of that particular mRNA in the presence of SOX was then calculated upon PAPII or PAPγ knockdown. The data are the mean±the standard error between experimental replicates (n = 3). (0.17 MB TIF) [file pbio.1000107.s002.tif]

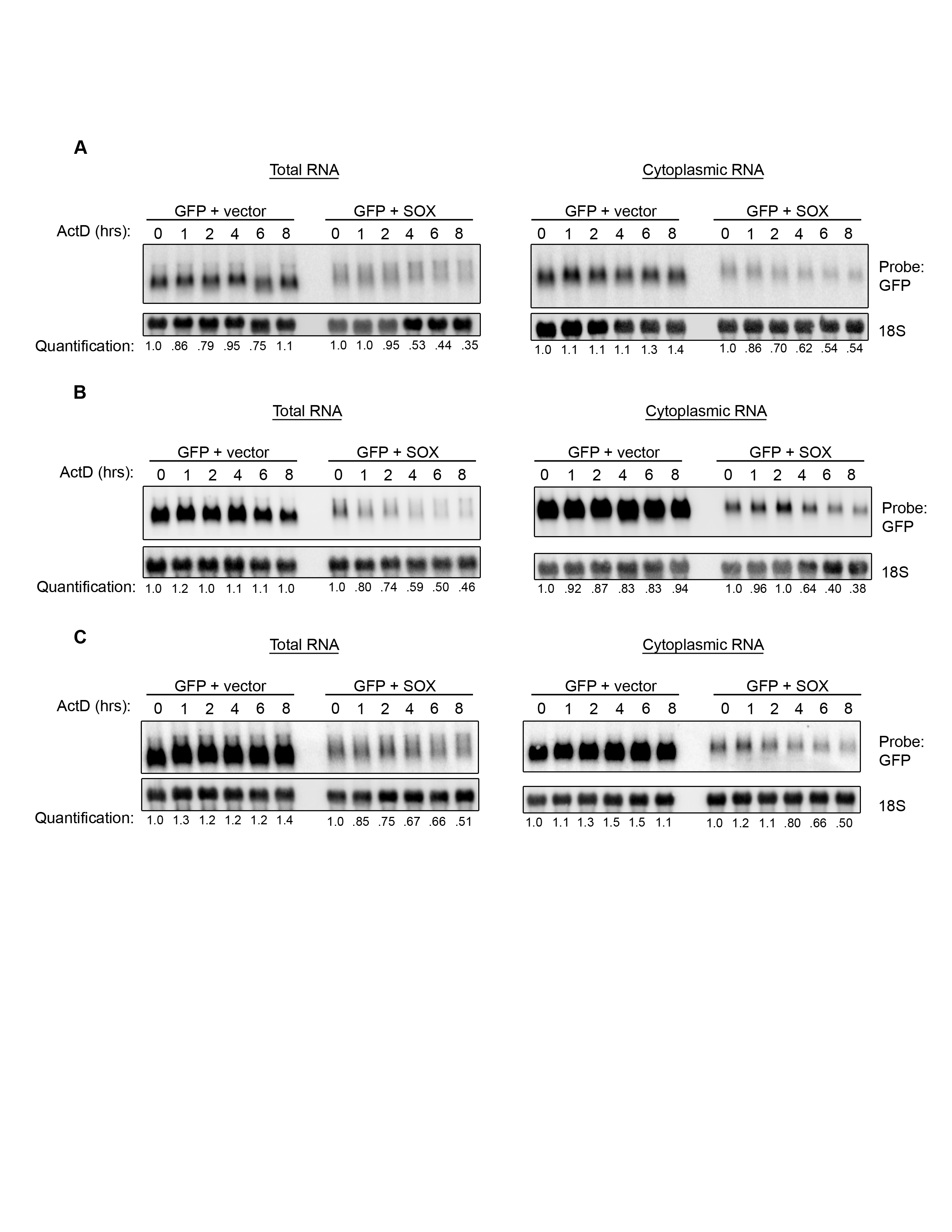

Supplement: Figure S3 — Gels showing half-life measurements of GFP mRNA in the cytoplasm of HEK 293T cells either in the absence or presence of SOX. HEK 293T cells were transfected with GFP alone (100 ng) or together with a SOX expression plasmid (200 ng). Twenty-four hours post-transfection, cells were treated with 1 µg/ml actinomycin D for the indicated time to halt transcription. Cytoplasmic RNA was then extracted and northern blotted with GFP and 18S probes. (8.45 MB TIF) [file pbio.1000107.s003.tif]

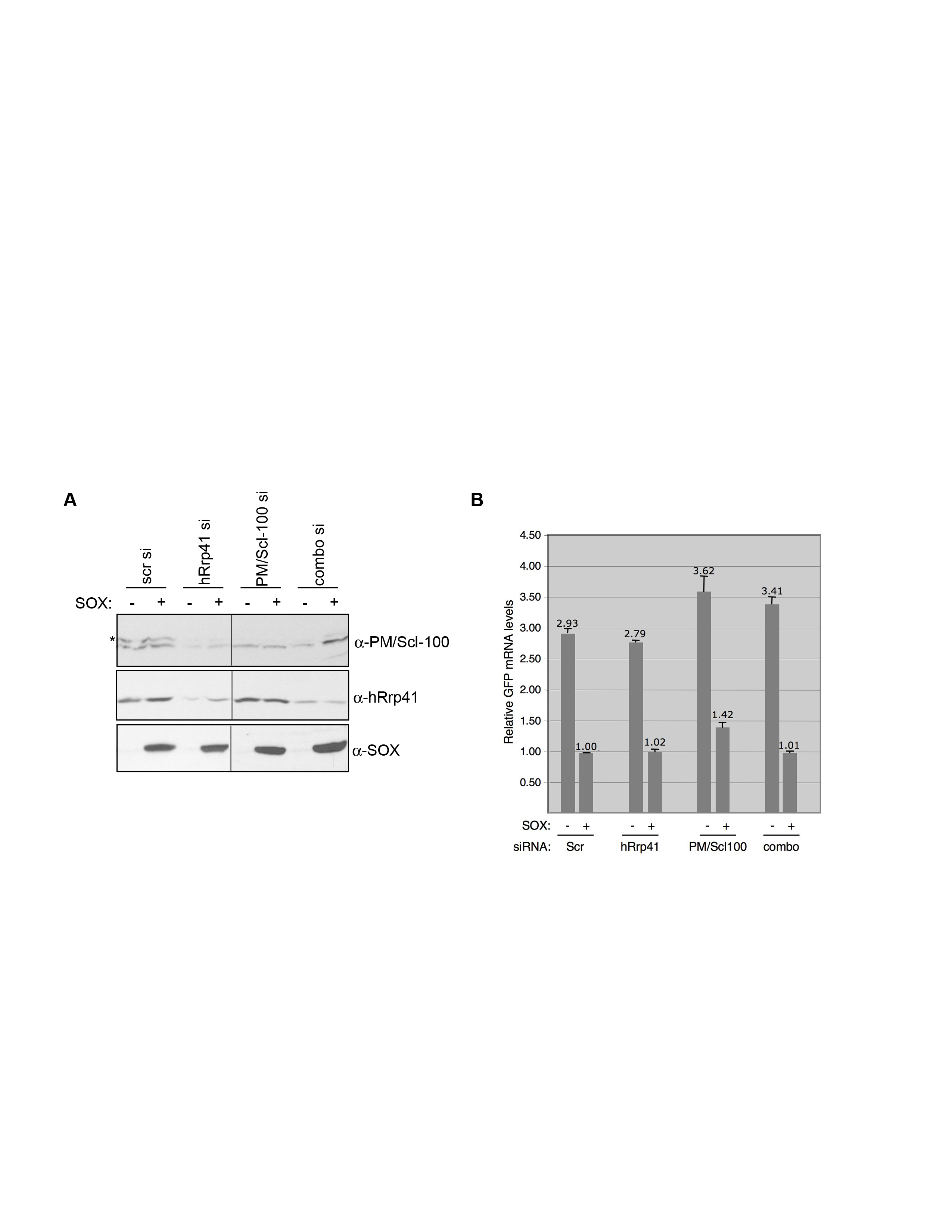

Supplement: Figure S4 — The exosome does not play an essential role in SOX-induced mRNA destruction. HEK 293T cells were transfected with hRrp41 or PM/Scl-100 (hRrp6) duplex siRNA oligos or a combination of hRrp41+PM/Scl-100 oligos, or a nonspecific control siRNA oligo (scr si). Twenty-four hours after the siRNA transfection, the cells were transfected with the indicated DNA plasmid(s) expressing GFP±SOX; each sample was split in half and, 72 h later, either harvested for protein and immunoblotted with polyclonal hRrp41 or PM/Scl-100 antibodies to measure the efficiency of siRNA-mediated knockdown (A), or harvested for total RNA and subjected to quantitative real-time PCR using GFP specific primers (B). As reported previously, Rrp41 knockdown leads to destabilization of other exosome components including PM/Scl-100 [57]. The asterisk in (A) marks the location of the PM/Scl-100 protein. Lines through gels indicate where an intervening lane was cropped out of the image. (0.97 MB TIF) [file pbio.1000107.s004.tif]

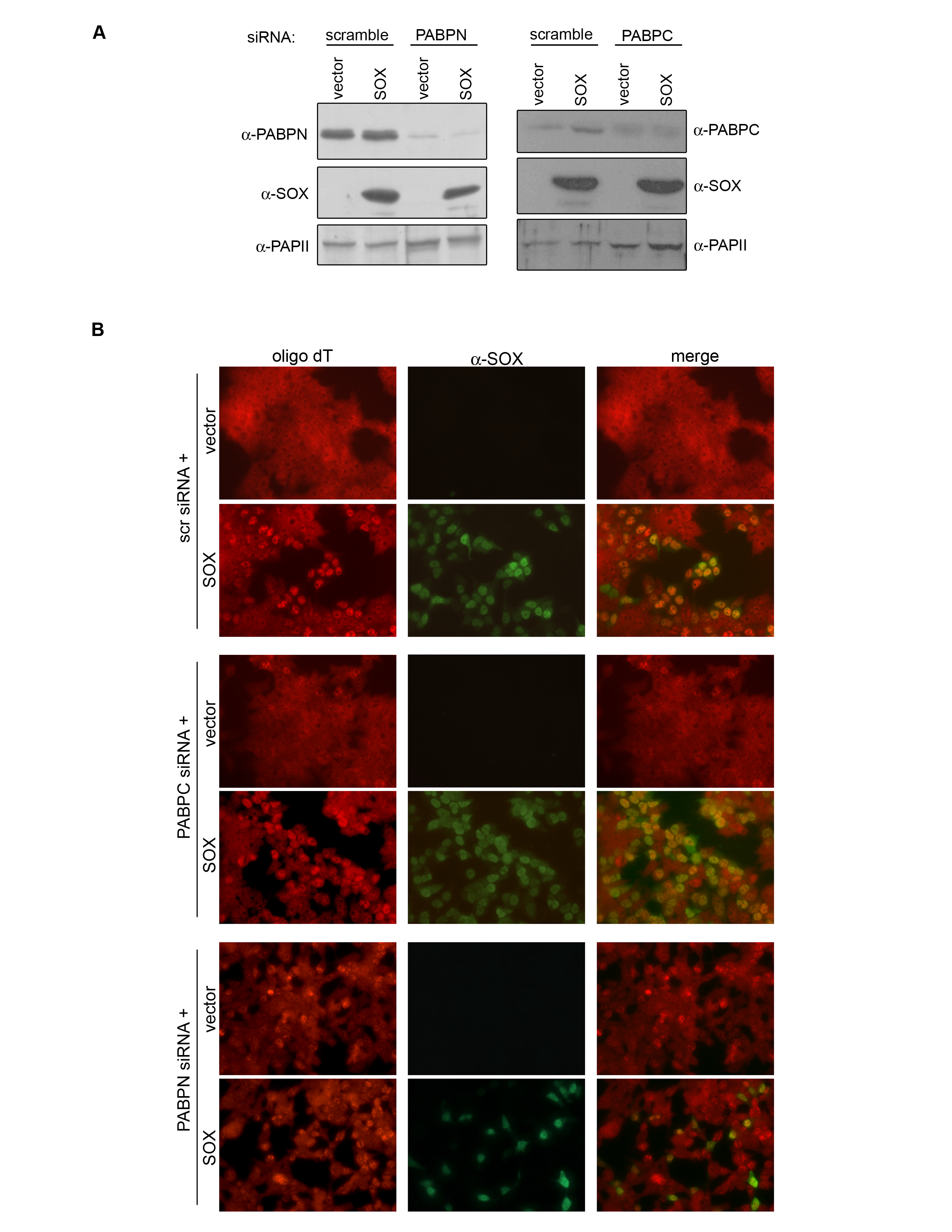

Supplement: Figure S5 — Oligo(dT) in situ hybridization in SOX-expressing cells following PABPC or PABPN knockdown. HEK 293T cells were transfected twice sequentially with either scramble control duplex siRNA oligos, or PABPC or PABPN duplex siRNA oligos. Twenty-four hours after the last siRNA transfection, the cells were split 1∶2 and transfected with empty vector or a SOX expression plasmid. Twenty-four hours later, half the samples were harvested for protein and western blotted for PABPC and PABPN to gauge the level of knockdown, as well as PAPII (as a loading control) and SOX (A). The other half of the samples were subjected to oligo(dT) in situ hybridization (left) and IFA with SOX antibodies (center) to monitor the ability of SOX to promote hyperadenylation of endogenous mRNAs in the absence of PABPC or PABPN (B). The right panels represent a merge of the oligo dT and SOX images. (4.19 MB TIF) [file pbio.1000107.s005.tif]

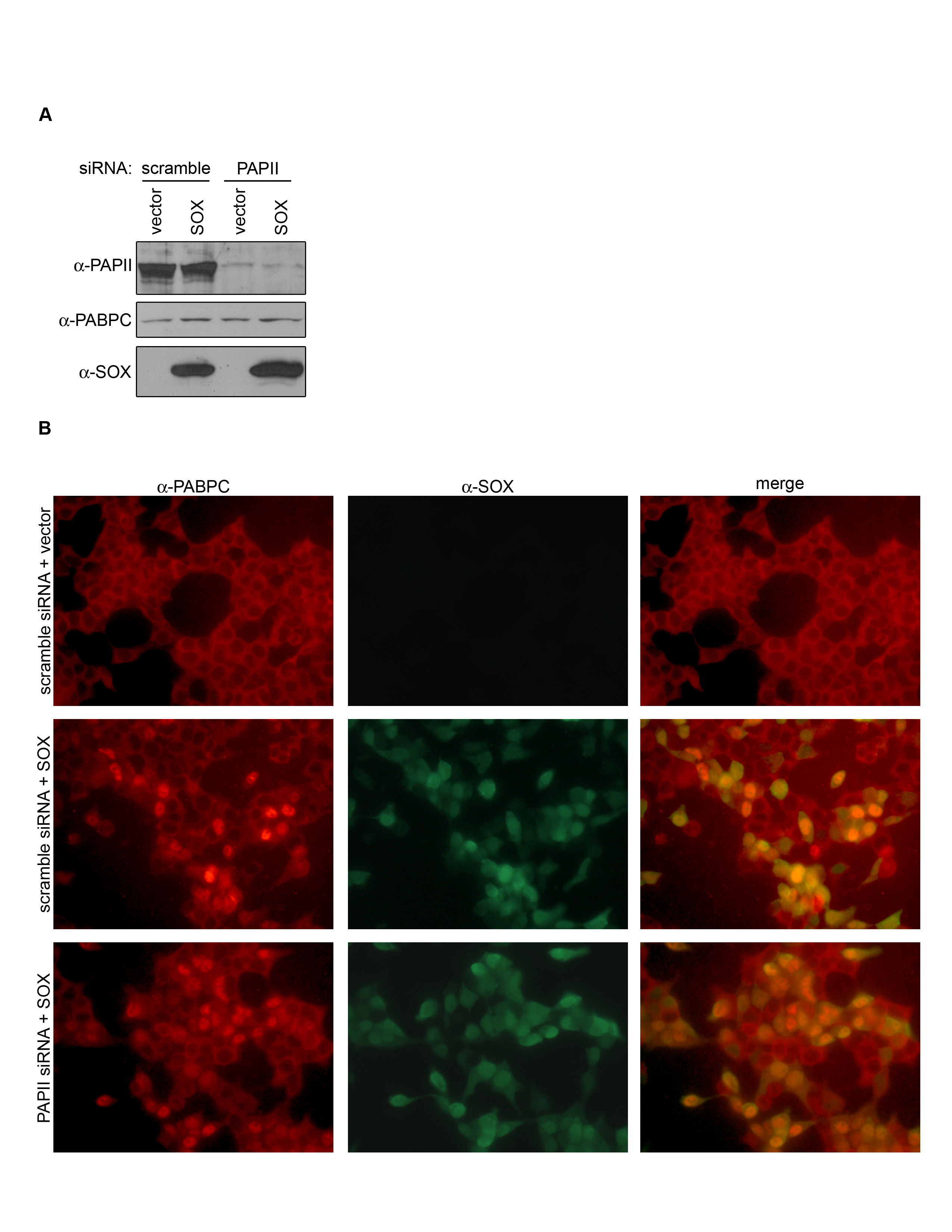

Supplement: Figure S6 — SOX-induced nuclear import of PABPC is not an indirect result of hyperadenylation. HEK 293T cells were transfected twice sequentially with either scramble control duplex siRNA oligos or PAPII siRNA oligos (to block hyperadenylation). Twenty-four hours after the last siRNA transfection, the cells were split 1∶2 and transfected with empty vector or a SOX expression plasmid. Twenty-four hours later, half the samples were harvested for protein and western blotted for PAPII to gauge the level of knockdown, as well as PABPC (as a loading control) and SOX (A). The other half of the samples were subjected to immunofluorescence analysis with PABPC (left) and SOX (center) antibodies to monitor the ability of SOX to promote import of endogenous PABPC in the absence of hyperadenylation (B). The right panels represent a merge of the PABPC and SOX images. (4.63 MB TIF) [file pbio.1000107.s006.tif]

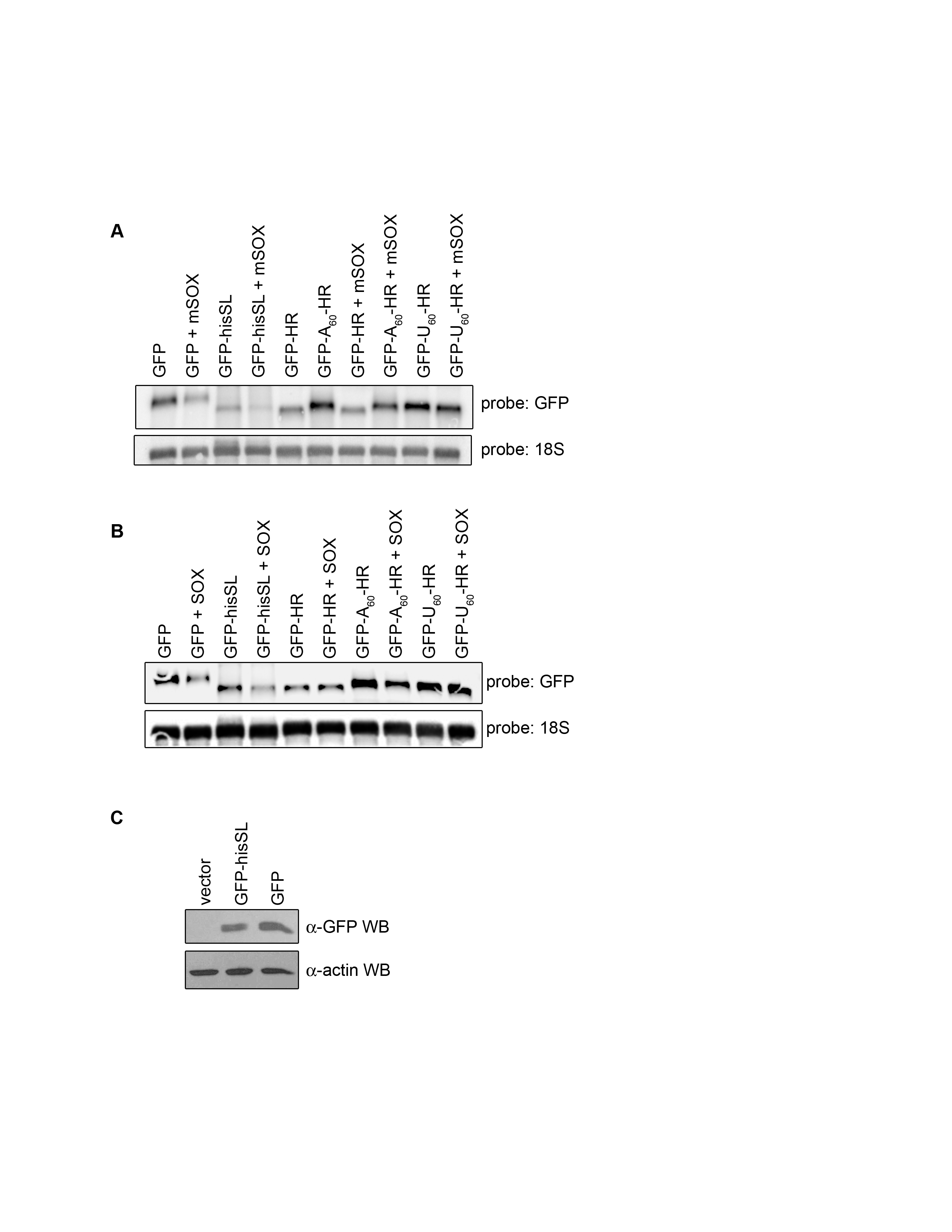

Supplement: Figure S7 — Degradation of GFP-HR and GFP-hisSL mRNAs by KSHV and MHV68 SOX. (A and B) HEK 293T cells were transfected with each of the indicated GFP plasmids alone or together with KSHV SOX (A) or the MHV68 SOX homolog (mSOX [B]) at a 1∶2 ratio. Total RNA was harvested from each sample 24 h post-transfection and northern blotted with GFP and 18S probes. (C) HEK 293T cells were transfected with the indicated plasmid (WT GFP or GFP terminating with the histone SL and termination sequences (GFP-hisSL); 24 h later, protein was harvested and western blotted with GFP antibodies to compare the level of protein expression from the GFP-hisSL construct relative to WT GFP. (0.48 MB TIF) [file pbio.1000107.s007.tif]

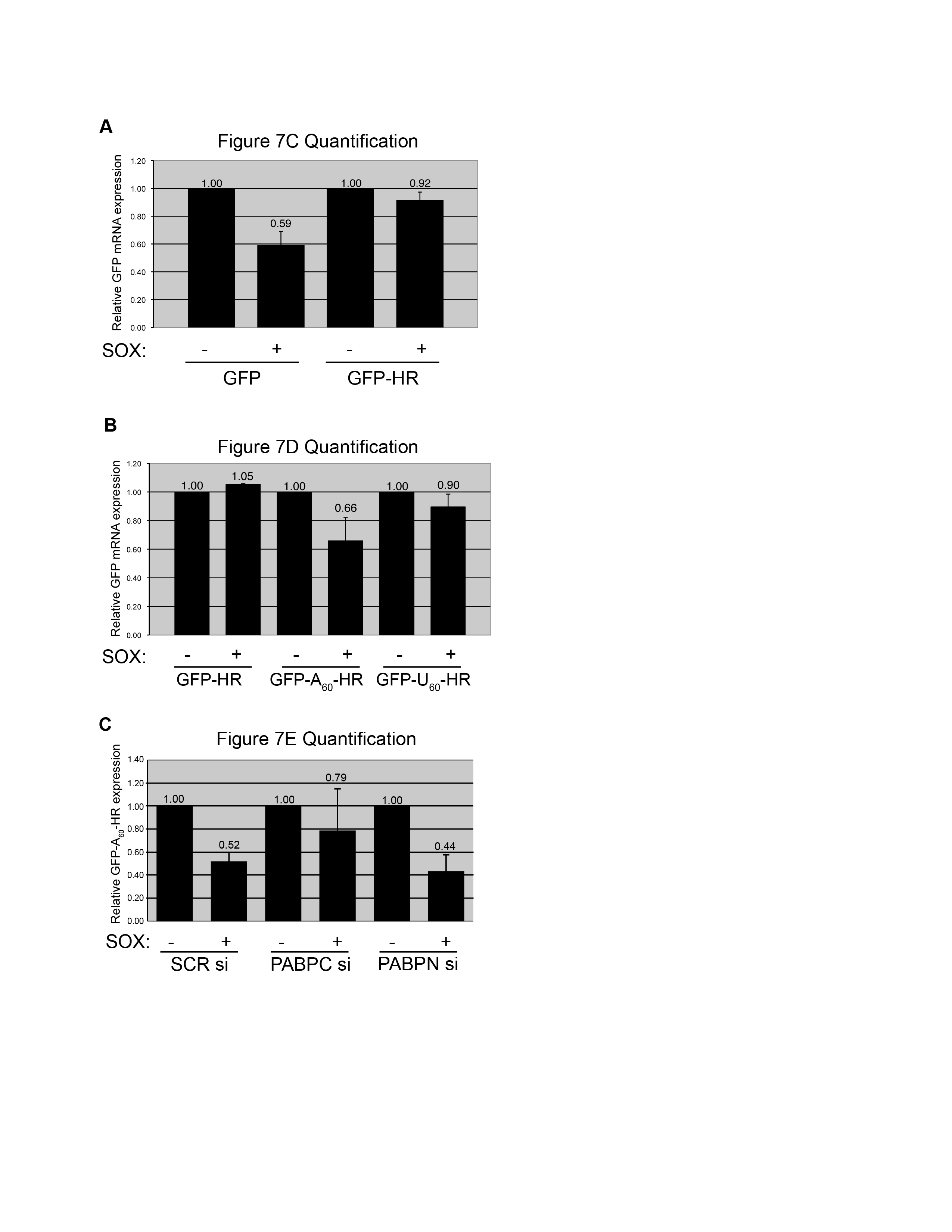

Supplement: Figure S8 — Quantification of SOX-induced mRNA turnover shown in Figure 6. (A and B) Quantification of SOX-induced turnover of GFP mRNA in the presence or absence of a poly(A) tail. HEK 293T cells were transfected with the indicated wild-type or ribozyme-terminating GFP construct in the presence or absence of SOX. Total RNA was harvested 24 h post-transfection and northern blotted with GFP and 18S probes. The level of each GFP or GFP-HR mRNA in the absence of SOX was set to 1.0 after normalization to 18S rRNA, and the corresponding level of that particular mRNA in the presence of SOX was then calculated. The data are the means±the standard error between experimental replicates (n = 4 for [A], n = 3 for [B]). (C) Quantification of the contribution of PABPC and PABPN towards GFP-A60-HR mRNA destruction by SOX. HEK 293T cells were transfected twice sequentially with PABPC or PABPN duplex siRNA oligos or nonspecific control siRNA oligos (scramble si). Twenty-four hours after the final siRNA transfection, the cells were transfected with the GFP-A60-HR plasmid alone or together with a SOX expression plasmid. Twenty-four hours later, the cells were harvested and subjected to northern blot analysis. Quantification was performed as described above (n = 3). (0.25 MB TIF) [file pbio.1000107.s008.tif]
